# ESDBO: A Multi-Strategy Enhanced Dung Beetle Optimization Algorithm for Urban Path Planning of UGV

**DOI:** 10.3390/s26030930

**Published:** 2026-02-01

**Authors:** Chenhui Wei, Zhifang Wei, Yanlan Li, Jie Cui, Yanfei Su

**Affiliations:** 1 School of Mechanical and Electrical Engineering, North University of China, Taiyuan 030051, China; b20240115@st.nuc.edu.cn; 2 College of Innovation and Entrepreneurship, North University of China, Taiyuan 030051, China; 20050168@nuc.edu.cn; 3 Department of Criminal Science and Technology, Shanxi Police College, Taiyuan 030051, China; cjzsj912@163.com; 4 North China Vehicle Research Institute, Beijing 100072, China; bq201syf@163.com

**Keywords:** dung beetle optimization algorithm, sine mapping, adaptive information volatilization mutation strategy, multi-mechanism co-evolution strategy, path planning

## Abstract

In the complex urban path planning of unmanned ground vehicles (UGVs), the dung beetle optimization (DBO) algorithm is widely used due to its simple structure and fast convergence speed. However, it still has the disadvantages of poor convergence accuracy and is easy to fall into a local optimum. To solve these problems, this paper proposes a multi-strategy enhanced DBO algorithm (ESDBO). Firstly, sine mapping is introduced in the population initialization stage to enhance solution diversity. Secondly, an adaptive information volatilization mutation strategy is proposed, which dynamically balances the convergence and global search ability. Finally, a multi-mechanism co-evolution strategy is designed, which significantly improves the local search ability and stability. Through ablation experiments and CEC2017 benchmark tests, the optimization ability of the proposed strategy and the convergence accuracy and stability of ESDBO are verified. Further path planning experiments are carried out on the public Random MAPF benchmark map. The results show that ESDBO can generate global optimal paths with short path length, few turns, and high safety margin on different obstacle densities and map scales. The algorithm provides an efficient and reliable solution for autonomous navigation in complex urban environments.

## 1. Introduction

With the rapid development of unmanned and intelligent technologies, the unmanned ground vehicle (UGV) has been widely used in intelligent transportation, disaster relief, environmental detection, and logistics distribution. With the advantages of high mobility, flexibility, low cost, and high efficiency, UGVs have gradually become an indispensable part of intelligent travel and service systems [1].

The urban environment has the characteristics of an irregular road network and dense distribution of obstacles. The path planning needs to comprehensively optimize the path length, smoothness, and safety margin under the feasibility conditions. This poses a higher challenge to UGV path planning [2,3,4]. In this case, path planning is usually described as a high-dimensional, nonlinear, and strongly constrained global optimization problem. Therefore, the development of a safe, adaptive, and efficient path planning method has become a key scientific and engineering problem to be solved in this field [5].

Traditional deterministic path planning algorithms include the Dijkstra algorithm [6], artificial potential field method [7], random tree techniques [8], A* search algorithm [9], and bi-level programming method [10]. In such a scenario, the traditional algorithms have difficulty fully considering the path characteristics in complex environments, resulting in high computational complexity, sensitivity to environmental noise, and limited flexibility. This makes it challenging to achieve high-quality solutions and stable performance at the same time. In contrast, the meta-heuristic algorithm has become an effective tool for high-dimensional, nonlinear, and multi-objective optimization due to its non-gradient, strong global search ability, and strong adaptability [11]. Over the past decade, meta-heuristic algorithms, such as the Particle Swarm Optimization (PSO) [12], Fruit Fly Optimization Algorithm (FOA) [13], Ant Colony Optimization Algorithm (ACA) [14], Artificial Bee Colony Algorithm (ABC) [15], Dung Beetle Optimizer Algorithm (DBO) [16] and Flower Pollination Algorithm (FPA) [17], have been extensively applied to path planning tasks. Despite their simplicity and ease of implementation, meta-heuristic planners may still suffer from slow convergence, premature convergence, and an exploration–exploitation imbalance in constrained scenarios [18]. Nevertheless, they remain one of the mainstream approaches for UGV path planning in complex environments.

In recent years, hybrid heuristics and multi-objective evolutionary algorithms have been widely adopted to improve robustness and solution diversity, such as PSO–GA hybrids [19] and decomposition-based MOEA [20,21]. These methods enhance multi-objective trade-offs by integrating complementary exploration–exploitation mechanisms. However, in high-dimensional or complex constrained scenarios, they are still prone to local optima [22]. These challenges have prompted researchers to explore bio-inspired algorithms that enhance adaptability and search diversity.

In addition to the improvement centered on the optimization algorithm, recent UGV path planning research is also moving along a complementary route. It includes task-driven collaboration [23], personalized constraint modeling in a specific environment [24], decomposition-based evolutionary algorithm architecture [25], and dynamic feasible motion generation with post-processing [26]. These directions together indicate that a robust and efficient global optimizer is still a basic and reusable building block under specific task settings.

Under the research background outlined above, this paper focuses on the offline global path planning problem of a single UGV in a known static environment. Its core aim is to construct a robust optimizer to deal with the feasible region search problem under high-dimensional, nonlinear, strong constraint and multi-objective conditions. The search performance, convergence stability, and solution quality of the optimization algorithm are strengthened, which lays a foundation for the subsequent trajectory feasibility test, the fusion of kinematic constraints, and the integration of online or distributed planning frameworks.

Against this backdrop, Xue and Shen (2023) proposed the DBO. Inspired by five natural behaviors of dung beetles—ball rolling, dancing, foraging, stealing, and reproduction—this algorithm constructs an optimization framework combining global search with local exploitation [16]. It demonstrates high convergence accuracy and stability in both benchmark tests and engineering optimization. It is worth noting that the location update mechanism of DBO reflects the tendency to avoid high-cost areas. Therefore, when the objective function of path planning contains obstacle penalty or safety constraints, DBO is more likely to generate candidate paths that meet the requirements of feasibility and safety margin, which is suitable for constrained path planning scenarios. However, in high-dimensional constrained search spaces such as UGV path planning, DBO may still have problems, such as insufficient initialization diversity and insufficient exploration–exploitation balance, and can easily fall into a local optimum in the later stage [27].

Consequently, a variety of DBO variants have been developed to improve exploration–exploitation balance and mitigate premature convergence. Li et al. proposed IDBO by integrating cubic-chaotic initialization, adaptive t-distribution perturbation, and cooperative local search, achieving faster and more accurate UAV 3D path planning [28]. Zu et al. developed QHDBO with quantum-driven t-distribution mutation to enhance population diversity and suppress premature convergence [29]. Jin et al. introduced a Tent chaotic map to construct Tent-DBO for underwater manipulator motion planning, improving initial distribution uniformity and obstacle-avoidance success [30]. Wu et al. proposed IMDBO for UAV path planning by incorporating multiple global search operators, yielding better smoothness, convergence efficiency, and robustness [31]. For swarm coordination, Huang et al. presented EDBO with hierarchical elite information sharing to strengthen global exploration without sacrificing local convergence [32]. Zheng et al. proposed BMDBO by combining adaptive parameter control and Lévy flight, improving convergence accuracy and stability on CEC benchmarks [33].

Although the improved DBO variants have made some progress in convergence performance and robustness, it has been found that they are often only enhanced for the individual defects above and are not fully applicable to high-dimensional and strictly limited path planning. Therefore, in view of the three limitations of the DBO algorithm above, this paper proposes an enhanced DBO algorithm framework (ESDBO) that integrates multiple strategies. The framework can increase the diversity of the population when the population is initialized, dynamically control the search process in the iterative process, manage the population structure, and avoid the problem of falling into a local optimum in the later stage. The effectiveness of the algorithm is verified by ablation and interaction analysis and comprehensive simulation experiments.

The innovations and main contributions of this paper are as follows:

- A multi-strategy enhanced DBO algorithm framework is designed.
- The initialization based on sine mapping is combined to enhance the coverage and uniformity of the early population and reduce the initial deviation. An adaptive information volatilization mutation strategy is designed. The strategy dynamically balances exploration and exploitation to alleviate stagnation in the middle and late stages.
- A multi-mechanism co-evolutionary strategy is proposed. By jointly adjusting the search boundaries and population structure, it enhances late-stage refinement and boundary convergence, thereby improving convergence accuracy and solution stability.
- A verifiable chain of evidence is provided, including ablation studies, sensitivity analysis, and synergistic effect analysis. Through statistical tests on the CEC2017 benchmark test set, the advantages of the framework are further verified.
- A multi-objective fitness function integrating path length, smoothness, and safety is constructed. ESDBO is evaluated on public MAPF grid maps of different sizes and obstacle densities. The results show that the proposed framework can consistently generate high-quality feasible paths and has good engineering applicability.

The structure of this paper is as follows. In Section 2, the basic DBO model is presented. In Section 3, an improved DBO algorithm (ESDBO) is suggested. In Section 4, the performance of the ESDBO is evaluated through simulation results. The ESDBO is used for the path planning of the UGV in Section 5. Finally, the conclusions of the paper are summed up in Section 6.

## 2. The Theory of the DBO Algorithm

The DBO algorithm, introduced in 2022, is a heuristic optimization algorithm based on the collective foraging behavior of dung beetles [34]. This algorithm mainly models five key behaviors of dung beetles during the collection, transportation, and utilization of dung balls, including ball rolling, dancing, foraging, stealing, and reproduction. Specifically, under the guidance of a celestial body, dung beetles use “ball rolling behavior” to push dung balls from their initial positions to new safe positions, and use “dancing behavior” at the appropriate time to avoid potential threats and obstacles, thus expanding their search scope. Throughout this process, different dung beetles choose suitable dung balls for “reproduction behavior” and “stealing behavior” according to criteria such as the quality or size of the dung balls, while the hatched minor dung beetles perform “foraging behavior” around the dung balls.

### 2.1. Ball Rolling and Dancing Behavior

**Figure 1:**
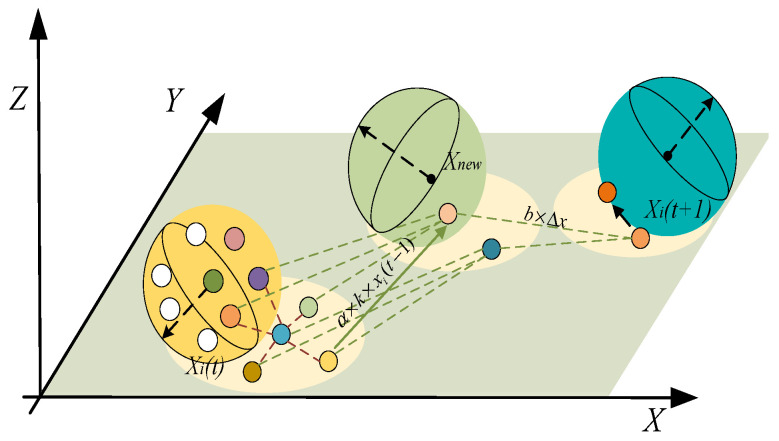
Schematic of the ball-rolling position update.

In nature, dung beetles roll dung balls guided by celestial bodies, enabling them to maintain a stable rolling direction while continuously correcting deviations caused by environmental disturbances. Their primary objective is to push the dung ball away from unsafe or unfavorable positions, reflecting a risk-averse movement strategy. Consequently, the rolling behavior in DBO is modeled as a constrained rolling process guided by external environmental cues (celestial bodies), wherein directional stability is maintained by celestial information while risk avoidance is achieved by introducing the globally most unfavorable position. This contrasts with the attraction to globally optimal positions found in other swarm intelligence algorithms, such as particle swarm optimization or ant colony optimization. The position update Equations (1) and (2) are defined as follows:

(1) $$x_{i}(t+1)=x_{i}(t)+\alpha \times k\times x_{i}(t-1)+b\times \Delta x$$

(2) $$\Delta x=\left|x_{i}(t)-X^{w}\right|$$

where $x_{i}(t+1)$ represents the location of the i-th dung beetle at t+1 iteration. The number of iterations is t, and the position of the i-th dung beetle at the t-th iteration is marked by $x_{i}(t)$. α can have values of either −1 or 1. k is a coefficient of deflection that falls in [0, 0.2]. The i-th location of the dung beetle at the t−1-th iteration is denoted by $x_{i}(t-1)$. b is a constant within the interval [0, 1]. Δx is the information increment simulating light intensity. $X^{w}$ is the global worst position. Figure 1 shows the position updating schematic during the rolling phase.

$X^{w}$ is the global worst individual position in the current iteration population. Its mathematical definition can be expressed as follows:

(3) $$X^{w}=x_{i_{w}}(t),\;i_{w}=\arg\underset{i\in \{1,\ldots ,N\}}{\max}f(x_{i}(t))$$

where *N* is the population size, and *f*(*x*) is the corresponding fitness. Its core idea is to take the global worst solution as the reference point. By measuring the distance between the individual and the global worst solution, the search step size and disturbance intensity are dynamically adjusted, so that the individual has stronger exploration ability when away from the bad solution area, thereby improving the overall search diversity and robustness of the algorithm.

When the dung beetle encounters predators or obstacles during the ball rolling process, the dung beetle uses “dancing behavior” to quickly adjust the direction and get rid of environmental restrictions. This underscores that dancing behavior does not represent an independent search phase. It is another movement mode when the ball rolling dung beetle needs to correct the direction. DBO uses a tangent-based function to simulate this dance behavior, which introduces a nonlinear angular perturbation to expand the local search range, where θ denotes the random deflection angle at which the dance behavior is triggered. Therefore, the location of the beetle update Equation (4) may be written as follows:

(4) $$x_{i}(t+1)=x_{i}(t)+\tan(\theta )\left|x_{i}(t)-x_{i}(t-1)\right|$$

where θ is the angle of deflection in the interval [0,π], and the position is not updated when θ is 0, π/2, or π.

It should be noted that Equations (1) and (4) do not compete or interfere with one another, as only one update rule is applied to the rolling individual during each iteration, thereby ensuring the consistency and coherence of the state update process.

### 2.2. Reproduction Behavior

The reproduction of dung beetles is an important ecological activity in their lives. Specifically, dung beetles roll the dung balls to a safe location, dig into the soil to bury them underground, and internally spawn. The boundary equations of the spawning region, given by Equations (5) and (6), are defined as follows:

(5) $$Lb^{*}=\max(X^{*}\times (1-R),Lb)$$

(6) $$Ub^{*}=\min(X^{*}\times (1+R),Ub)$$

where $Lb^{*}$ and $Ub^{*}$ are used to define the lower and upper boundaries of the spawning region, respectively. $X^{*}$ is the current local optimal spawning position. $R=1-t/T_{\max}$, where $T_{\max}$ is the maximum number of iterations and t is the current iteration number. The lower and upper boundaries of the search space are denoted by Lb and Ub. With the increase in the number t, the range of the upper and lower boundaries of the spawning region contracts progressively, enhancing computational efficiency.

Based on the upper and lower boundary information of the spawning region, the dung beetles transport the dung balls carrying their eggs to the designated location. Under the DBO, it is assumed that each dung beetle produces only one egg per iteration. During the iteration process, the spawning region is dynamically changed. The position update formula Equation (7) is given as follows:

(7) $$B_{i}(t+1)=X^{*}+b_{1}\times (B_{i}(t)-Lb^{*})+b_{2}\times (B_{i}(t)-Ub^{*})$$

where $B_{i}(t)$ denotes the current locally optimal reference position corresponding to the i-th dung beetle at the t-th iteration, which serves as the center of the dung ball area during the reproduction behavior. $b_{1}$ and $b_{2}$ are two independent random vectors with a size of 1×D, where D represents the dimension of the problem.

### 2.3. Foraging Behavior

The foraging behavior of dung beetles can be used as a model to explain the activity of minor dung beetles. After maturation in the egg, they begin to forage for energy and nutrients that are essential for their development. Minor dung beetles rely on their sense of smell to perceive and identify the best foraging region. The boundary Equations (8) and (9) for the foraging region are defined as follows:

(8) $$Lb^{b}=\max(X^{b}\times (1-R),Lb)$$

(9) $$Ub^{b}=\min(X^{b}\times (1-R),Ub)$$

where $Lb^{b}$ and $Ub^{b}$ are the lower and upper boundaries of the globally optimal foraging region. $X^{b}$ is the current global position for foraging at the current iteration. The position update Equation (10) for juvenile dung beetles is defined as follows:

(10) $$x_{i}(t+1)=x_{i}(t)+C_{1}\times (x_{i}(t)-Lb^{b})+C_{2}\times (x_{i}(t)-Ub^{b})$$

where $x_{i}(t)$ is the position information of the i-th stealing dung beetle at the t-th iteration. $C_{1}$ is a randomly generated number following a normal distribution. $C_{2}$ is a random vector within the range [0, 1].

### 2.4. Stealing Behavior

The stealing behavior of dung beetles simulates the natural phenomenon that some dung beetles steal dung balls from other dung beetles. The location update formula for stealing dung beetles Equation (11) is given as follows:

(11) $$x_{i}(t+1)=X^{b}+S\times g\times (\left|x_{i}(t)-X*\right|+\left|x_{i}(t)-X^{b}\right|)$$

where $x_{i}(t)$ is the position information of the i-th stealing dung beetle at the t-th iteration. S is a predefined constant. g is a random vector following a normal distribution with dimensions 1×D. $\left|x_{i}(t)-X*\right|$ is the difference between the current position and the locally optimal position. $\left|x_{i}(t)-X^{b}\right|$ is the difference between the current position and the global best position.

Algorithm 1 presents the basic structure of the DBO algorithm. The first line represents the random initialization of the dung beetle population and calculates the fitness of each individual based on the objective function. Lines 9 through 25 describe the position update process of ball rolling, dancing, foraging, stealing, and reproduction of dung beetles. Line 25 represents the termination condition evaluation statement.
**Algorithm 1** Dung Beetle Optimizer (DBO)**Input**: The parameters of DBO, such as population size (*N*), max iterations (*T*_max_), solving dimension (*D*)**Output**: Global best Dung Beetle *X^b^*1:Initializing *n* dung beetles in search space *x_i_*; and evaluate fitness values for all dung beetles *f_i_* (*i* = 1, 2, …, *N*)2:Determine the global best position *X^b^* and global worst position *X^w^*.3:Set control parameters: *k*, *b*, *S*.4:*for t* = 1 to *T*_max_
**do**5: **for** each dung beetle *x_i_ do*6:  **Ball-rolling behavior and Dancing Behavior**
7:  δ = rand (1);8:  **if**
*δ* < 0.9 **then** // stable rolling mode9:   Update the position of the dung beetle of Ball-Rolling Behavior by Equation (1)10:  **else //** direction correction mode11:   Update the position of the dung beetle of Dancing Behavior by Equation (4).12:  **end if**
13:  **Reproductive behavior**
14:   Update the upper and lower bounds of the boundary region by Equations (5) and (6).15:   Update the position of the dung beetle of Reproductive Behavior by Equation (7).16:  **Foraging Behavior**
17:   Update the upper and lower boundaries of feeding regions by Equations (8) and (9).18:   Update the position of the dung beetle of Foraging Behavior by Equation (10).19:  **Stealing Behavior**
20:   Update the position of the dung beetle of Stealing Behavior by Equation (11).21: **end for**
22:  Update global best solution *X^b^* and worst solution *X^w^*.23: **if** the termination condition is met, **then**24:  **Break**
25: **end if**
26:**end for**27:**return** Global best Dung Beetle *X^b^*

## 3. The Proposed Method of the ESDBO

ESDBO is a multi-strategy enhanced DBO algorithm integrating sine mapping, adaptive information volatilization mutation strategy, and multi-mechanism co-evolution strategy. These strategies are not simply a combination of discrete modules but targeted integration into population initialization, ball rolling, foraging, reproduction, and stealing behavior. It effectively promotes early exploration, solves the imbalance between global exploration and local development, and improves convergence stability. Unless otherwise stated, all the fixed parameters associated with the algorithm are consistent with the parameters outlined in Section 2, “DBO algorithm theory”.

### 3.1. Sine Mapping

**Figure 2:**
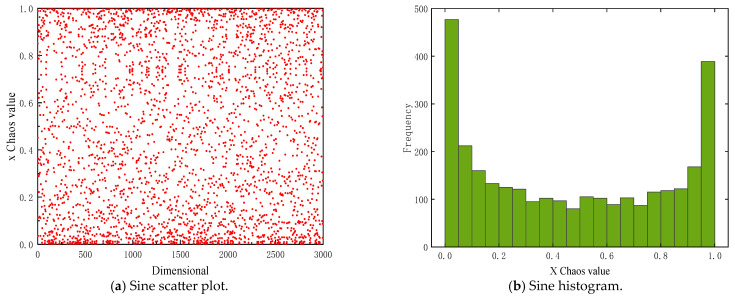
Distribution characteristics of Sine mapping. (**a**) Sine scatter plot. (**b**) Sine histogram.

Chaotic mapping is a mathematical model in nonlinear dynamical systems, which shows random characteristics while following deterministic mathematical rules. Sine mapping is a chaotic mapping that dynamically evolves state variables through the application of sine functions [35]. The initial value of the sine mapping shows a high sensitivity to the state variables so that it can generate complex and diverse solution structures in the solution space. In this paper, sine mapping is introduced into the DBO algorithm to initialize the entire dung beetle population, ensure effective global search coverage, reduce the risk of early aggregation, and enhance search diversity. The corresponding mathematical expression Equation (12) is given as follows:

(12) $$x_{n+1}\;=r\times sin(\pi x_{n}\;)$$

where r is a chaotic control parameter within the range [0, 1]. $x_{n}$ represents the state variable at the n-th iteration. Figure 2 shows the scatter plot and histogram of the Sine mapping. As shown in Figure 2, the Sine mapping falls within the [0, 1] range, displaying high irregularity and strong self-similarity. The histogram shows the phenomenon of higher density at both ends and lower density in the middle, indicating that the Sine mapping tends to concentrate values at the boundaries during its initial value evolution, and the middle region is relatively sparse, demonstrating a typical chaotic distribution characteristic. This mechanism mainly affects the early population initialization stage of DBO.

Specifically, each data point in Figure 2a corresponds to the state value generated by the sine mapping of a given iterative step size. Figure 2b statistically summarizes the frequency of these state values of the fixed iteration step size. The visualization shows the time evolution and overall distribution characteristics of sinusoidal chaotic sequences.

### 3.2. Adaptive Information Volatilization Mutation Strategy

**Figure 3:**
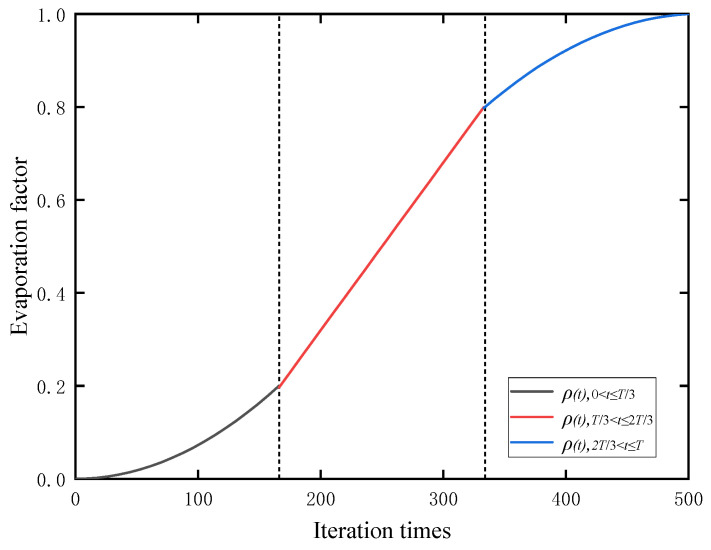
A three-stage change diagram of the information volatilization factor for adaptive mutation.

In this paper, an adaptive information volatilization mutation strategy is proposed, which directly acts on the position update formula of “ball rolling behavior”. The strategy designs an information volatilization mechanism, which integrates the volatilization factor into the information increment, and dynamically attenuates and adjusts the historical information in the path search, thereby reducing the risk of falling into a local optimum and mid-term stagnation, and inhibiting premature convergence [36]. The corresponding formula is given as follows (Equation (13)):

(13) $$\rho (t)=\left\{\begin{array}{ll} \rho_{min}+K_{1}\times t^{2}, & 0\leq t\leq T/3\\ \rho_{min}/2+\rho_{max}/2+K_{2}\times t, & T/3<t\leq 2T/3\\ \rho_{\max}\;+K_{3}\times {\left(t-T\right)}^{2}, & 2T/3<t\leq T \end{array}\right.$$

where $\rho_{\max}$ is the maximum volatilization factor, and $\rho_{\min}$ is the minimum volatilization factor. t is the current iteration time. $K_{1},K_{2},K_{3}$ are growth coefficients. *T* is the maximum number of iterations. Figure 3 shows the variation of the ρ(t) function, where ρ values increase as the number of iterations grows. In the early iterations, the volatilization rate exhibits a slowly increasing trend, preserving more celestial body information and expanding the global search space. In the middle iteration phase, the volatilization rate increases linearly, ensuring a smooth transition of the iteration process and enhancing solution accuracy. In the late iteration stage, the volatilization rate slows down to a certain extent, maintaining the pheromone concentration of high-quality paths and preventing them from being diluted too quickly.

The mutation strategy mainly addresses the balance between using the current optimal solution and exploring new solutions. The randomness is increased in the search process, so that the dung beetle can jump out of the local optima, explore the unknown regions in the solution space, and improve the global search ability of the algorithm. Therefore, this paper introduces a disturbance obeying a Gaussian distribution to simulate the mutation behavior. The corresponding formula is given as follows (Equation (14)):

(14) $$f(\mu ,\sigma )=(1/\sqrt{2\pi \sigma^{2}}\;)\times \exp(-{\left(x-\mu \right)}^{2\;}/2\sigma^{2})$$

where μ is the mean value. σ is the standard deviation, and σ changes with iteration. The corresponding formula is defined as follows (Equation (15)):

(15) $$\sigma (t)=\sigma_{end}-(\sigma_{end}\;-\sigma_{start\;})\times t/T\;$$

where $\sigma_{start\;}$ and $\sigma_{end\;}$ are the initial and final standard deviations. In the beginning, a relatively large σ is set to strengthen stochastic exploration. In the later stage, the standard deviation is gradually reduced to limit the disturbance amplitude. As iterations progress, the standard deviation is gradually decreased to constrain the perturbation.

Therefore, the position update formula of the rolling behavior of the dung beetle is improved by using the adaptive information volatilization mutation strategy. The corresponding position updating formula is given as follows (Equation (16)):

(16) $$x_{i}\;(t+1)=x_{i}\;(t)+\alpha \times k\times x_{i\;}(t-1)+(1-\rho (t))\times b\times \Delta x+\gamma \times f(\mu ,\sigma )$$

where $x_{i}(t)$ is the position information of the i-th dung beetle at iteration t. ρ is the information volatilization factor within the range [0, 1], and γ is the mutation intensity control factor. f(μ,σ) is a random function following a Gaussian distribution. The equation incorporates the previous positions of the dung beetle, the worst solution location, and stochastic mutations to adaptively update the solution position, ensuring a balance between local exploration and global exploration.

### 3.3. Multi-Mechanism Co-Evolution Strategy

The multi-mechanism co-evolution strategy is inspired by the foraging behavior of microorganisms such as Escherichia coli, including chemotaxis, reproduction, and migration stages [37]. Inspired by this, the co-evolutionary framework constructed in this paper contains four complementary mechanisms: adaptive boundary adjustment, local chemotactic foraging, survival-of-the-fittest reproduction, and migration mechanisms. This strategy mainly strengthens the later development and improves the stability of the solution.

#### 3.3.1. Adaptive Boundary Adjustment Mechanism

In the DBO, the dynamic contraction factor R decreases linearly, resulting in the boundaries for dung beetle foraging and reproduction to shrink monotonically, reducing the adaptability of the iteration process. To address these issues, this paper proposes an adaptive boundary adjustment mechanism based on sinusoidal fluctuation. This mechanism replaces the boundary contraction formula of reproduction and foraging behavior in the original DBO so that the search space boundaries in DBO can be dynamically expanded or shrunk as needed. The new region update mechanism is formulated as an equation as follows.

(17) $$Lb^{n}\;=max(X(t)\times (1-R_{g}),Lb^{n}\;)$$

(18) $$Ub^{n}\;=min(X(t)\times (1+R_{g}\;),Ub^{n}\;)$$

(19) $$R_{g}=1-t/T_{\max}+\lambda \sin(2\pi t/T_{\max})$$

where $Lb^{n}\;$ and $Ub^{n}$ are the dynamic lower and upper boundaries of the region. X(t) denotes the population state set composed of all individual positions *x*_*i*_(*t*), utilized for fitness evaluation, ranking, and determining global/local optimal solutions. $R_{g}$ is an adaptive operator. λ is the amplitude parameter. As t increases, $R_{g}$ overall declines but incorporates a sinusoidal periodic term, causing periodic oscillations during the descent process. This boundary oscillation allows the algorithm to jump out of the current contracted region, facilitating broader exploration and preventing the neglect of solutions near the boundaries.

#### 3.3.2. Local Chemotactic Foraging Mechanism

In the DBO foraging behavior, minor dung beetles forage around the global optimal region. However, their search lacks direction. This causes the individuals to walk randomly within the region without effectively converging to the optimal solution. To improve the foraging behavior and enable more effective local search, this paper introduces the chemotaxis search mechanism. This mechanism adds the refined optimization steps beyond the standard update, deeply explores the better solutions near the current solution, enhances the local search capability, and guides the algorithm more effectively toward the global optimum. The formulation of the local chemotactic foraging mechanism is presented in the formula as follows (Equation (20)):

(20) $$\left\{\begin{array}{l} x_{i}(t+1)=x_{i}(t)+\gamma [C_{1}\times (x_{i}(t)-Lb^{b})+C_{2}\times (x_{i}(t)-Ub^{b})]+C_{c}\;\times v_{i}/∥v_{i}∥\;\\ v_{i}=bestX-x_{i}(t)\\ bestX=\min[f(x_{i}(t))],\;x_{i}(t)\in [Lb^{b},Ub^{b}] \end{array}\right.$$

where $x_{i}(t)$ is the current individual position. γ is a chemotactic gain coefficient.

$C_{c}$ is the chemotaxis step size, which is proportional to the local foraging boundary scale, and $v_{i}/∥v_{i}∥$ is the unit direction vector, which ensures that the individual advances the unit step size in the direction of the optimal solution. $v_{i}$ is the direction in which the individual is moving toward the optimal solution. bestX is the current global optimal solution. $Lb^{b}$ and $Ub^{b}$ are the lower and upper bounds of the optimal foraging area, respectively. Young dung beetles can further optimize their position to prevent them from being trapped in local areas.

#### 3.3.3. Survival-of-the-Fittest Reproduction Mechanism

The DBO generates an oviposition site for each female dung beetle during the reproduction stage. But it lacks an explicit natural selection process. To improve the quality of the population, this paper proposes a breeding mechanism for the survival of the fittest and optimizes the population structure regularly during the reproduction stage. Specifically, the cumulative fitness of the population chemotaxis process is used as the index of breeding energy, and the individuals of the group are sorted according to the energy level: individuals with higher energy are selected to produce offspring to replace the inferior individuals. The new generation in the population consists of replicated elite individuals and new individuals generated through mutation. This reproduction mechanism can retain the high-quality solution of the dung beetle population, prevent the accumulation of poor solutions, and help to improve the stability and convergence speed of the algorithm. The equation for the survival-of-the-fittest reproduction mechanism is given in Equation (21) as follows:

(21) $$\left\{\begin{array}{ll} E_{i}(t)=\sum_{t=1}^{T_{c}}f(x_{i}(t)) & \\ \left[\sim ,idx]=Sort(\{E_{i}(t)\}\right) & \\ B_{idx}(t+1)=B_{n\text{-}idx}(i)+0.01\times N(0,1), & idx<n/2\\ B_{i}(t+1)=X^{*}+b_{1}\times (B_{i}(t)-Lb^{*})+b_{2}\times (B_{i}(t)-Ub^{*}), & idx\geq n/2 \end{array}\right.$$

where $T_{c}$ is the fitness evaluation window. $E_{i}(t)$ is the cumulative fitness value of the *i* dung beetle. Sort() is an ascending permutation function. idx(i) is the index sorted based on fitness value. N(0,1) is a normally distributed disturbance, which introduces small random perturbations to replicated individuals to prevent offspring from being identical to their parents, thereby avoiding population degradation. $b_{1}$ and $b_{2}$ represent two independent random vectors by size 1 × *D*. By employing a survival-of-the-fittest reproduction mechanism, the algorithm allocates more computational resources to higher fitness solutions, filtering out ineffective search directions. This enhances both the convergence speed and the reliability of the obtained solutions.

#### 3.3.4. Migration Mechanism

In the stealing phase of the DBO, a small probability is assigned to certain individuals to “migrate” out of the current region and be randomly reinitialized at other locations within the search space. This mechanism helps to alleviate the premature convergence caused by an excessive concentration of individuals. The formulation of the migration mechanism is given in formula Equation (22).

(22) $$x_{i}(t+1)=\left\{\begin{array}{ll} L+r_{i}(U-L)\sin(x_{i}(t)), & P<P_{ed}\\ k_{1}\times X^{b}+k_{2}\times S\times g\times (\left|x_{i}(t)-X*\right|+\left|x_{i}(t)-X^{b}\right|), & otherwise \end{array}\right.$$

where $r_{i}$ is a random variable. $k_{i}$ is an adaptive correction coefficient, $k_{1}=1-t^{3}/T^{3}$, $k_{2}=t^{3}/T^{3}$. Ped is the mutation probability.

### 3.4. Detailed Steps for the ESDBO

**Figure 4:**
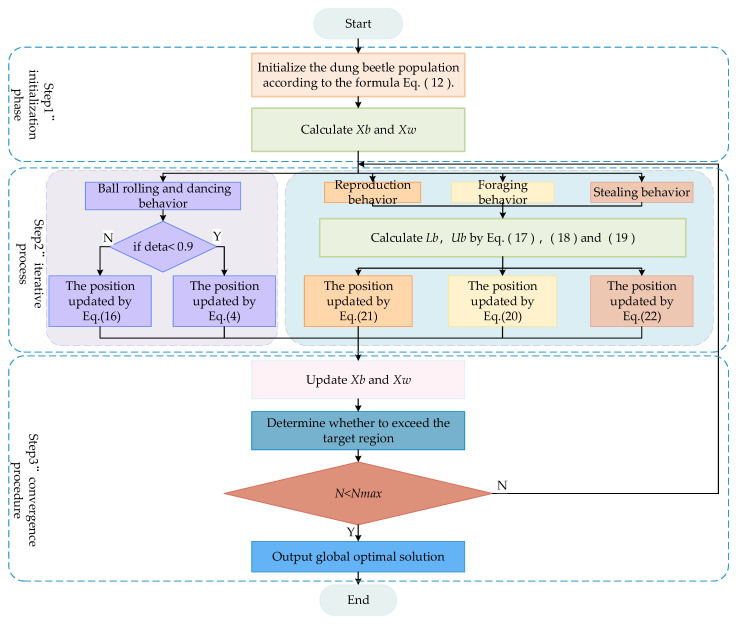
Workflow of ESDBO.

The flowchart of the ESDBO is shown in Figure 4. The pseudocode based on the ESDBO algorithm is presented in Algorithm 2.
**Algorithm 2** Improved Dung Beetle Optimization (ESDBO)**Input**: The parameters of the ESDBO, such as population size (*N*), max iterations (*T*_max_), solving dimension (*D*)**Output**: Global best Dung Beetle *X^b^*1:Using the **sine mapping**, initialize the positions *xi* of n dung beetles in the search space by Equation (12).2:Evaluate fitness values for all dung beetles *f_i_* (*i* = 1, 2, …, *N*).3:Determine the global best position *X^b^* and global worst position *X^w^*.4:**for** *t* = 1 to *T*_max_
**do**5: Calculate adaptive *ρ* and mutation factor *f* based on *t* by Equations (13)–(15).6: **for** each dung beetle *x_i_*
**do**7:  **Ball Rolling Behavior and Dancing Behavior**
8:  δ = rand (1);9:  **if**
δ < 0.9 **then** // stable rolling mode10:    Update the position of the dung beetle of Ball Rolling Behavior by Equation (16).11:   **else //** direction correction mode12:    Update the position of the dung beetle of Dancing Behavior by Equation (4).13:   **end if**
14:  Calculate the adaptive operator *R_g_* by Equation (19).15:  **Reproductive Behavior**
16:  Update the upper and lower bounds of the boundary region by Equations (17) and (18).17:  Update the position of the dung beetle of Reproductive Behavior by Equation (21).18:  **Foraging Behavior**
19:  Update the upper and lower boundaries of feeding regions by Equations (17) and (18).20:  Updated the position of the dung of Foraging Behavior by Equation (20).21:  **Stealing Behavior**
22:  Update the position of the dung beetle of Stealing Behavior by Equation (22).23: **end for**
24:  Update global best solution *X^b^* and worst solution *X^w^*.25: **if** the termination condition is met, **then**26:   **Break**
27: **end if**
28:**end for**29:**return** Global best Dung Beetle *X^b^*

### 3.5. Time Complexity Analysis

In order to evaluate the computational overhead introduced by the proposed mechanism, this section analyzes the time complexity of ESDBO, assuming that the population size is N, the problem dimension is D, and the maximum number of iterations is T. The computational cost of the original DBO algorithm is composed of initialization and D-dimensional position update per round. In each iteration, each needs to update its position in D dimensions. Therefore, the time complexity of the standard DBO is expressed as follows:

(23) $$O_{DBO}=O(T\cdot N\cdot D)$$

On this basis, the ESDBO algorithm introduces three enhancement strategies. The sine mapping strategy generates chaotic sequences and constructs N individuals with D dimensions. The one-time overhead is O(N⋅D), and it is only performed once in the initialization phase, which does not affect the asymptotic complexity of the iterative process. The adaptive information volatilization mutation mechanism needs to update the volatilization factor and inject the disturbance term during the iteration process. The essence is to perform D-dimensional vector operation on the population, which can be recorded as O(N⋅D) in each iteration in the worst case. The multi-mechanism co-evolutionary strategy includes fitness-based sorting and subsequent behavior updating. The complexity of the sorting stage is O(NlogN), while the boundary convergence, foraging, reproduction, and stealing are mainly based on D-dimensional vector calculation, and the single iteration cost is O(N⋅D). In this paper, the experimental setting usually satisfies D≫logN, so the dominant term is TND, and the overall complexity can be approximately written as O(T⋅N⋅D). The total time complexity of ESDBO can be expressed as follows:

(24) $$O_{ESDBO}=O(N\cdot D)+T(O(N\cdot D)+O(N\cdot D)+O(NlogN)+O(N\cdot D))\approx O(T\cdot N\cdot D)$$

The conclusion shows that the proposed enhancement mechanism mainly introduces constant level and sorting item overhead but does not change the same complexity order as the benchmark DBO, and the computational cost remains controllable.

## 4. Simulation Experiment and Analysis

All the experiments were conducted on a PC running Windows 11 (64-bit) with an Intel Core i5-9300H CPU and 16 GB RAM. The following aspects are mainly analyzed: (1) main parameter configurations, (2) ablation experiments, and (3) comparative analysis with other swarm intelligence algorithms.

### 4.1. Main Parameter Configuration and Sensitivity Analysis

#### 4.1.1. Main Parameter Configuration

In this paper, we evaluate ESDBO on 23 standard test functions [38] and the CEC2017 benchmark suite [39], which covers unimodal, multimodal, hybrid, and composite functions.

In the aspect of simulation experiment design, this paper sets up two kinds of test schemes. Firstly, three DBO variants are constructed based on the proposed enhancement mechanism and compared with the original DBO and ESDBO to quantitatively evaluate the contribution and mechanism of each enhancement strategy to the performance of the algorithm. Secondly, five typical swarm intelligence optimization algorithms are selected as baselines (PSO [40], WOA [41], GWO [42], WHO [43], and DBO), and two recently representative DBO improved algorithms (IDBO [29], TDBO [30]) are further introduced for horizontal comparison. All the test functions and parameter configurations are uniformly set according to the relevant literature to ensure the fairness, repeatability, and objectivity of the comparison.

Each algorithm runs 200 times independently. The population size is 30, and the number of iterations is 500. The average fitness value and standard deviation of each algorithm are calculated. All the parameters follow the reference settings and are summarized in Table 1.

#### 4.1.2. Parameter Sensitivity Analysis

In order to evaluate the robustness of ESDBO under parameter changes, this section uses a single-factor perturbation method. Sensitivity analysis of the volatility factor ρ and chemotaxis step length $C_{c}$ is conducted. While ensuring that other parameters are fixed, the target parameters are scaled to measure the change in performance. Specifically, the scaling factor of the volatilization factor is set to $S_{\rho }\in \left\{0.5,0.8,1,1.2\right\}$, and the scaling factor of the chemotaxis step is set to $S_{C_{c}}\in \left\{0.5,0.8,1,1.2,1.5\right\}$. Four representative test functions in CEC2017 are selected for testing, including unimodal function CEC-3, simple multimodal CEC-7, mixed function CEC-13, and composite function CEC-23. For each set of parameter configurations, each test function runs independently 200 times, and the mean ± standard deviation is counted to eliminate the influence of randomness.

**Figure 5:**
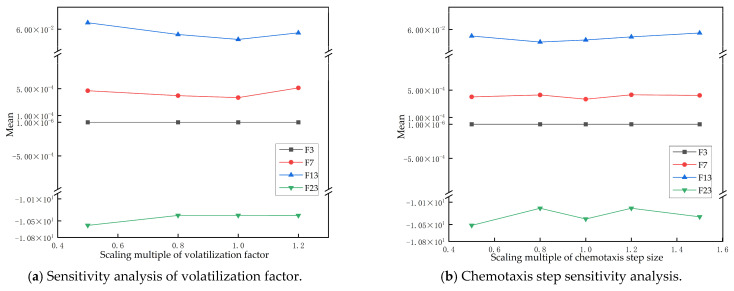
Sensitivity analysis of the ESDBO parameters.

As shown in Figure 5, when the volatilization factor ρ changes, F3 always maintains a very small order of magnitude close to 0, indicating that the solution accuracy of the algorithm on the single-peak problem is highly robust to this parameter. The fluctuation range of F23 is very small, showing good stability. Relatively speaking, there are some fluctuations in F7 and F13, but the overall trend is continuous and smooth, and there is no obvious instability. For the chemotaxis step $C_{c}$, the corresponding curves of each test function also maintain a small fluctuation and no abnormal transition, and the overall fluctuation range is more moderate than ρ. Overall, ESDBO shows limited performance fluctuation under the tested parameter scalings, suggesting acceptable robustness without extensive tuning.

### 4.2. Ablation Experiments

#### 4.2.1. Ablation Setup and Single-Mechanism Contribution

**Figure 6:**
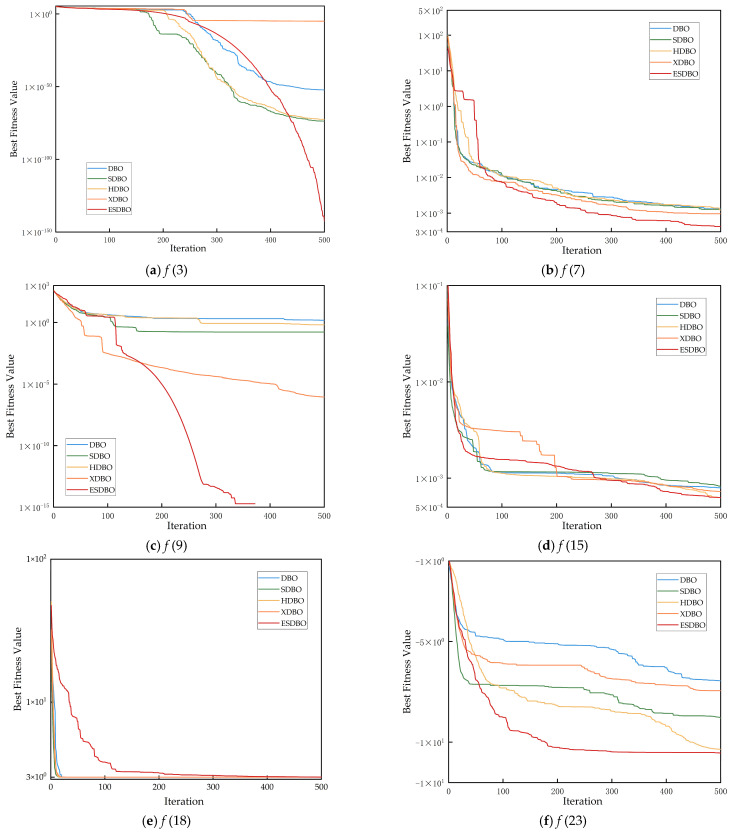
Ablation convergence curves on representative CEC2017 functions (F3, F7, F9, F15, F18, F23) for DBO and its variants.

In this section, this paper conducts a systematic analysis of the three key improvement strategies proposed, verifying their independent contributions and examining their synergistic effects when integrated. Based on these enhancement strategies, three algorithmic variants are derived: SDBO (Sine-based DBO), HDBO (adaptive information volatilization mutation-based DBO), and XDBO (multi-mechanism cooperative evolution-based DBO). These are compared with the original DBO and the ESDBO algorithms. Through comparative experiments on standard test functions, the impact of each mechanism on algorithm performance is systematically evaluated. Figure 6 displays partial convergence curves for different improvement strategies.

The convergence in Figure 6 reveals that the three improvement strategies play different but interrelated roles in the optimization process, showing obvious stage-complementary characteristics. SDBO decreases faster than DBO, HDBO, and XDBO in the initial stage of iteration (0~50 rounds). This phenomenon shows that the sine mapping strategy improves the population coverage and uniformity in the early stage of the search and alleviates the initial solution deviation. It is easier to quickly locate high-quality regions, which provide a solid foundation for global exploration in subsequent searches. HDBO shows a smoother downward trend in the middle and late stages of convergence. The adaptive information volatilization mechanism effectively balances global and local utilization. It alleviates the common “platform” phenomenon of DBO. It is the core driving force to maintain the optimization momentum of the algorithm in the middle and late iterations. In the later stage, XDBO obtains the highest accuracy and the most stable curve, indicating that the four-mechanism co-evolution strategy strengthens local refinement and boundary convergence and improves the robustness of the final solution. It is beneficial to generate high-quality feasible solutions that meet the requirements of path smoothness and safety margin.

The ESDBO, integrating the three strategies, always achieves lower fitness values with smaller fluctuations in all test functions and iterations. This shows that the three strategies form an indispensable nonlinear synergistic effect. As shown in Figure 6, ESDBO inherits the rapid decline characteristics of SDBO in the early stage, maintains a smoother and more continuous convergence trend of HDBO in the middle and late stages, and absorbs the high precision and high stability of XDBO in the later stage, thus realizing the full-stage closed-loop optimization of “early exploration, medium-term prevention of premature convergence, and later fine development”. Removing any strategy will lead to significant stage-specific degradation. Based on this, ESDBO enters the low-fitting region faster on the unimodal function and suppresses the premature convergence more effectively on the multimodal/composite function, thereby obtaining a consistently higher final accuracy.

#### 4.2.2. Synergy Verification and Interaction Analysis

**Figure 7:**
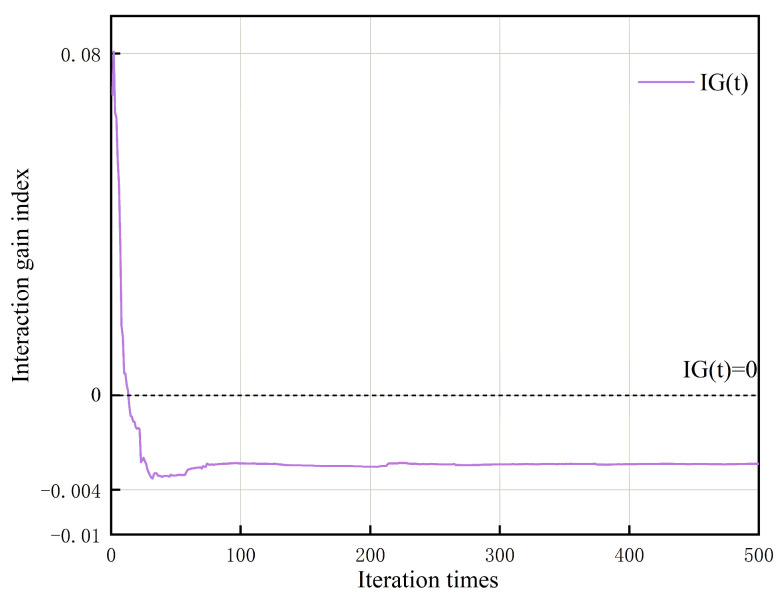
The interaction gain characteristic curve between the three improvement strategies.

The results of the ablation experiments show that each proposed enhancement mechanism promotes the optimization process through different behavioral effects, and their integration produces obvious synergistic effects rather than simple improvement accumulation. To quantify whether the fused strategy yields gains beyond single-strategy superposition, we introduce an interaction gain measure:

(25) $$IG(t)=3\times {\hat{J}}_{ESDBO}(t)-{\hat{J}}_{SDBO}(t)-{\hat{J}}_{HDBO}(t)-{\hat{J}}_{XDBO}(t)$$

where ${\hat{J}}_{SDBO}(t)$, ${\hat{J}}_{XDBO}(t)$, and ${\hat{J}}_{HDBO}(t)$ denote the average optimal fitness values of the three individual improvement algorithms at iteration t, while ${\hat{J}}_{ESDBO}(t)$ represents the fitness of the fusion algorithm. When IG(t)<0, it indicates that the performance of the integrated algorithm exceeds the expected linear sum of the three algorithms, signifying the presence of super-additive synergy. When IG(t)>0, it suggests the existence of redundancy or inhibitory effects among the strategies. Due to space constraints, this paper presents comprehensive interactive performance characteristics using only one representative class of test functions, as shown in Figure 7.

The experimental results indicate that during the mid-to-late stages, the interaction gains consistently remained negative, demonstrating that the combination mechanism operates through complementary interactions rather than simple additive effects. This validates the consistency between the interaction trends observed by the ensemble algorithm and the aforementioned qualitative analysis.

### 4.3. Comparative Analysis with Other Swarm Intelligence Algorithms

In order to validate the comprehensive performance and stability of the proposed ESDBO algorithm, simulation experiments were conducted based on 23 standard test functions from the CEC2017 benchmark dataset. The simulation comparison set retained a small number of typical traditional meta-heuristic algorithms (PSO, WOA, GWO, WHO) while incorporating recently proposed DBO variants (IDBO, TDBO). To maintain consistent experimental conditions, parameters including maximum iterations, population size, and test dimension align with those defined in Section 4.1.

**Figure 8:**
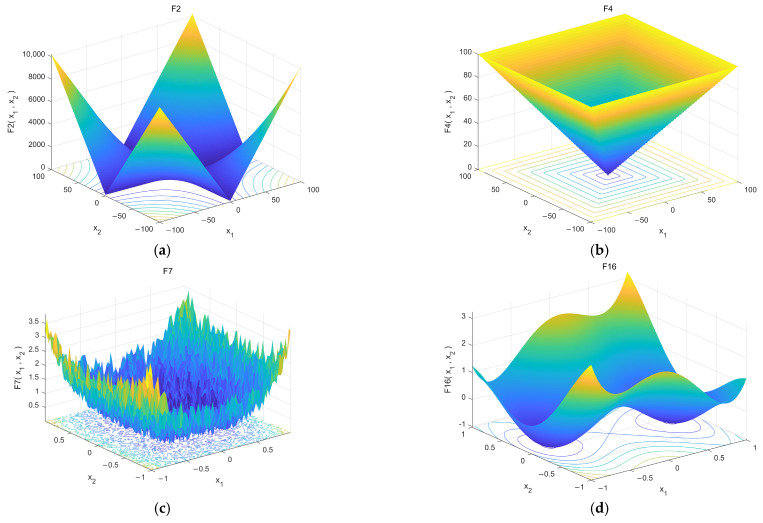
3D landscapes of representative CEC2017 benchmark functions.

Each test scenario is executed 200 times independently, and the mean and standard deviation of the fitness value are recorded. Figure 8 is a three-dimensional (3D) rendering of some benchmark functions. Table 2, Table 3, and Table 4 show the simulation results of different algorithms in 30-dimensional, 50-dimensional, and 100-dimensional spaces, respectively, and the optimal results are marked in bold. The Friedman test was used to obtain the average rank, and then the post hoc Wilcoxon signed rank test with Holm correction was performed (test level α = 0.05). The last row of the table reports the overall mean rank and Win/Tie/Loss count of the corrected comparison.

**Figure 9:**
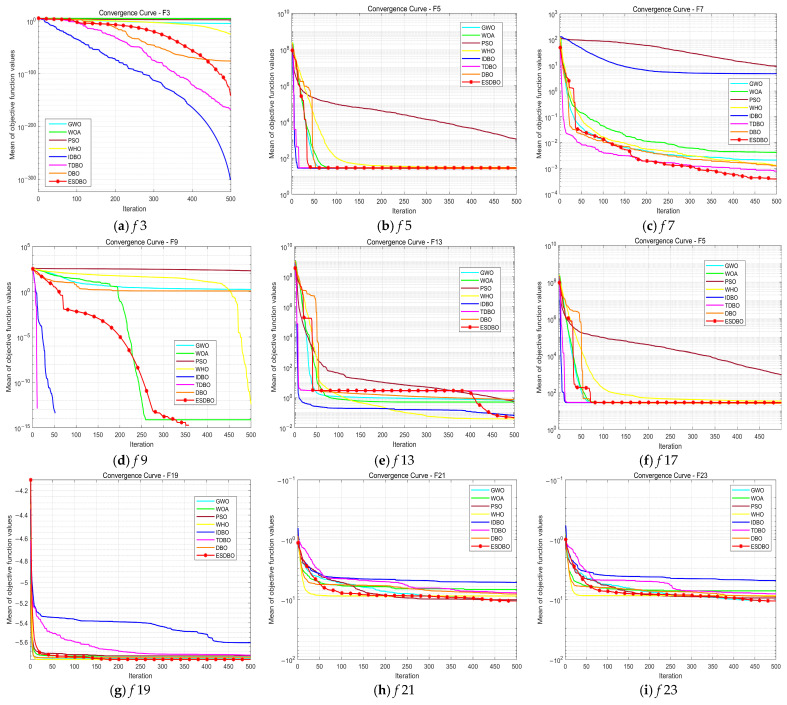
The convergence curve of ESDBO on the CEC2017 benchmark test function.

**Figure 10:**
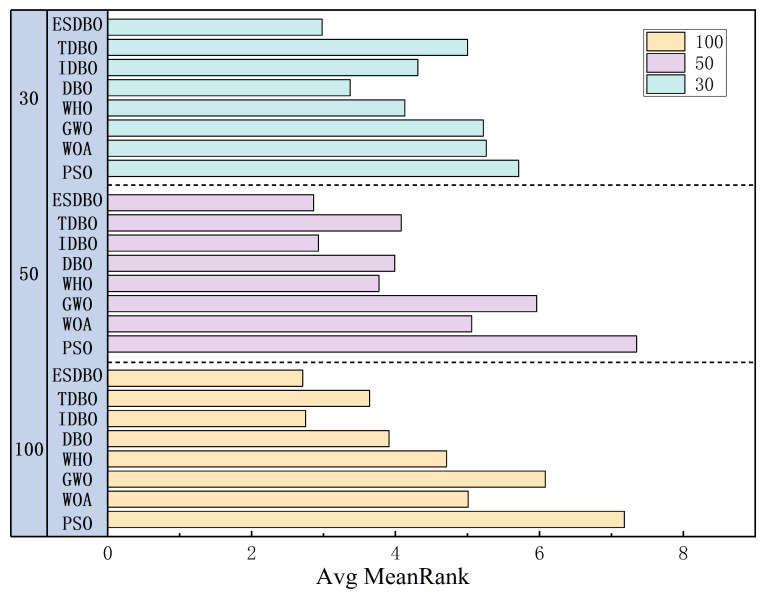
Average Friedman ranks of the compared algorithms (30D/50D/100D).

The results of Table 2, Table 3 and Table 4 and Figure 9 show that ESDBO is significantly better than traditional meta-heuristic algorithms (PSO, WOA, GWO, WHO) on most test functions. And it is still superior to the DBO family variants (DBO, IDBO, TDBO) in terms of accuracy and stability. Specifically, on the unimodal function (F1–F4), ESDBO achieves a near-zero fitness, and the error is in the order of 10^−70^, indicating fast and accurate convergence. On multimodal functions (F5~F10), ESDBO shows smaller dispersion and more reliable exploration–utilization balance as the dimension increases. On the fixed dimension functions (F14~F23), ESDBO achieves the best average performance on 7 of the 10 fixed dimension functions. While maintaining a small standard deviation, the fitness value is one or more orders of magnitude lower than that of other algorithms. Consistently, as shown in Figure 10, the Friedman ranking puts ESDBO at the top of the eight algorithms, Avg MeanRank = 2.98 (30D), 2.86 (50D), and 2.71 (100D), confirming its overall robustness and effectiveness.

In addition, the last row of each table summarizes the post hoc comparison results of Holm correction between ESDBO and each comparison algorithm (significance level α = 0.05). Specifically, after performing the overall Friedman test to verify whether there are significant differences between multiple algorithms, we performed a paired Wilcoxon signed-rank test on ESDBO and each baseline and used Holm’s stepwise decline correction to control the total type I error rate. The symbols “+”, “−”, and “=” represent statistically significant advantages, disadvantages, or no significant differences compared with the corresponding algorithms, respectively. If p_holm < 0.05, the performance difference is statistically significant, while p_holm ≥ 0.05 indicates that the performance difference is not statistically significant. The test results show that ESDBO has statistically significant advantages on most test functions, especially compared with the classical meta-heuristic algorithm. Compared with the DBO series of improved algorithms, it maintains a more balanced and stable leading edge. In summary, ESDBO shows stable advantages and good generalization ability under different optimized landscapes and dimension settings.

## 5. Path Planning of UGV Using the Proposed ESDBO

In Section 4, the ESDBO showed better convergence speed, solution accuracy, stability, and generalization ability. It is worth emphasizing that the inherent “avoiding inferior” search tendency of the DBO algorithm is naturally consistent with the problem structure of the path planning of UGV safety priority, strong constraint of the feasible region, and multi-objective trade-off. Therefore, based on the public benchmark map dataset, this section further evaluates the path feasibility, smoothness, and operational efficiency of ESDBO under different obstacle distributions [44]. The simulation results show that the ESDBO algorithm has great potential in solving complex optimization problems.

### 5.1. Construction of Urban Environment Model

Given that urban roads are generally flat and obstacles are mostly distributed in a regular pattern, this paper employs a grid-based approach to construct the map environment. This method discretizes the urban space, enabling clearer and more manageable representation of roads and obstacles, thereby providing an ideal environmental foundation for path planning and environmental perception.

In order to enhance the scientific rigor and representativeness of the path planning experiment, this paper uses the public random map in the MAPF test map data set. The data set has standardized obstacle distribution and statistical randomness, so it is widely used to verify the performance of multi-agent path planning algorithms. Specifically, two canonical graphs, Random-32-32-10 and Random-32-32-20, are selected as the basic environment. We constructed nine scenarios: 10 × 10-{10,20,30}, 15 × 15-{10,20,30}, and 32 × 32-{10,20,30}, where the first two numbers represent the map size and the last number represents the obstacle coverage (%). This “scale × density” scene design can systematically analyze the adaptability of the algorithm under different spatial scales and environmental complexity.

All the maps above employ a 2D grid model, where passable areas are marked as “0” and obstacles as “1”. White squares denote passable areas, while black squares represent obstacles. Each grid cell is uniquely indexed by a serial number. The coordinate system is defined via the coordinate-index mapping formula Equation (26).

(26) $$\left\{\begin{array}{l} x_{i}=a((i\mathrm{mod}N_{x})-0.5)\\ y_{i}=a(N_{y}+0.5-\left\lceil i/N_{y}\right\rceil ) \end{array}\right.$$

where $\left(x_{i},y_{i}\right)$ is the position coordinate of the i-th grid. $N_{x}$ and $N_{y}$ are the number of grids in the row and column directions, respectively.

### 5.2. Multi-Factor Fitness Function

In a complex urban environment, the quality of UGV path planning is not only related to the path length but also needs to consider the safety of driving. Therefore, based on the three evaluation indexes of path length, safety, and smoothness, this paper constructs a multi-factor fitness function of path planning.

#### 5.2.1. Path Length

Path length is a key metric for evaluating path quality. When planning routes, UGVs should prioritize minimizing path length to reduce travel time and energy consumption. The path length is defined as follows:

(27) $$L(P)=\sum_{i=1}^{n-1}d(P_{i}\;,P_{i+1\;})\;$$

(28) $$d(p_{i},\;p_{i+1})=\sqrt{{\left(x_{i+1}-x_{i}\right)}^{2}+{\left(y_{i+1}-y_{i}\right)}^{2}}$$

where n is the number of grids on the path. $P_{i}$ is the i th grid on the path, and $\left(x_{i},y_{i}\right)$ is the coordinate value of $P_{i}$. $d(P_{i},P_{i+1})$ is the Euclidean distance from $P_{i}$ to $P_{i+1}$.

#### 5.2.2. Safety

The safety index quantifies the ability of the UGV to avoid obstacles and mitigate environmental threats. Adding this item allows the optimizer to weigh the risks caused by path length and smoothness. The safety index is defined as follows:

(29) $$S(P)=\sum_{i=1}^{N}\phi (p_{i})\;$$

where $P\;=\left\{p_{1},p_{2},\ldots ,p_{N}\right\}$ denotes the sequence of discrete path nodes. The local safety function $\phi (p_{i})$ is defined hierarchically based on the spatial relationship between nodes and obstacles:

(30) $$\phi (p_{i})=\left\{\begin{array}{l} C_{col},\;If\;p_{i}\;falls\;into\;the\;obstacle\;unit\;or\;crosses\;the\;boundary\\ C_{nei},\;If\;there\;is\;an\;obstacle\;in\;the\;p_{i}\;neighborhood\\ C_{free},\;f\;p_{i}\;and\;its\;neighborhood\;are\;free\;elements \end{array}\right.$$

#### 5.2.3. Smoothness

Smoothness quantifies the continuity of the path by penalizing sharp turns or abrupt changes in direction or speed and is usually evaluated by path curvature. The smoothness index Equation (31) is defined as follows:

(31) $$G(P)=\sum_{i=2}^{n-1}\;\kappa_{i}^{2}$$

(32) $$\kappa_{i}=\left|\left(x_{i+1\;}-x_{i\;})(y_{i\;}-y_{i-1\;})-(x_{i\;}-x_{i-1})(y_{i+1}\;-y_{i\;}\right)\right|/\sqrt{{\left(x_{i+1\;}-x_{i\;}\right)}^{2}-{\left(y_{i+1}\;-y_{i\;}\right)}^{2}}$$

where $\kappa_{i}$ is the curvature of the path at point $P_{i}$.

In practical applications, each metric typically carries a distinct physical meaning and priority. Considering the inherent trade-offs among these objectives, a weighted combination of the three evaluation criteria is employed to construct the overall fitness function, as defined in Equation (33):

(33) $$J(P)=k_{L}\times L(P)+k_{s}\times S(P)+k_{G}\times G(P)$$

where $k_{L}$, $k_{s}$, and $k_{G}$ are the weight coefficients of path length, safety, and smoothness, respectively. In this way, the weight combination can flexibly handle the relationship between path length, safety, and smoothness indicators. The weight coefficients in this paper are set to 0.4, 0.4, and 0.2 by default.

### 5.3. Analysis of Simulation Results of the Path Planning of UGV

In order to verify the effectiveness of the algorithm, the path planning simulation is carried out on the MATLAB R2019b platform. Nine benchmark map scenarios defined in Section 5.1 are constructed. The starting position and the target position are set in the upper left corner and the lower right corner of each map, respectively. During the experiment, the population size is set to 50, the maximum number of iterations is set to 1000, and the fitness function is set to the content defined in Section 5.2. In order to ensure the reliability and validity of the statistical results, each algorithm runs independently 30 times.

**Figure 11:**
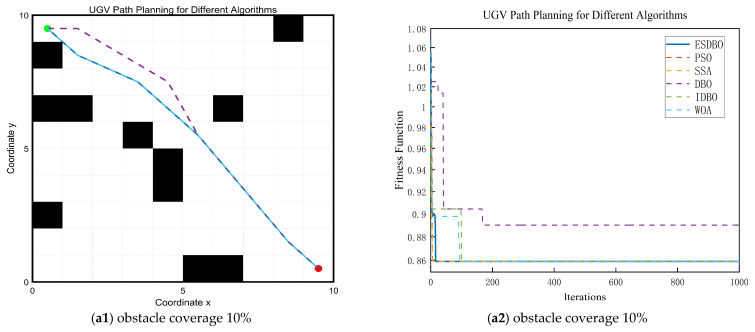

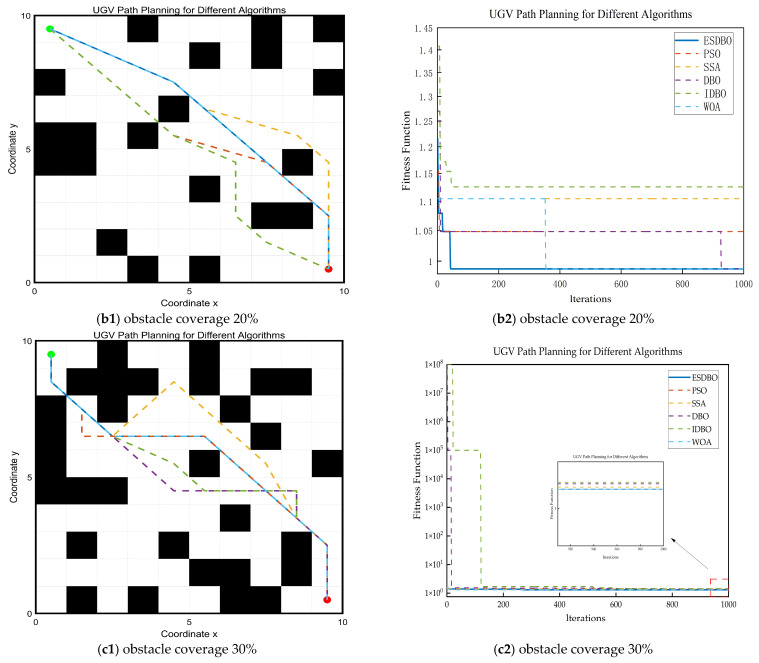
Path planning results and convergence curves on a 10 × 10 grid map.

**Figure 12:**
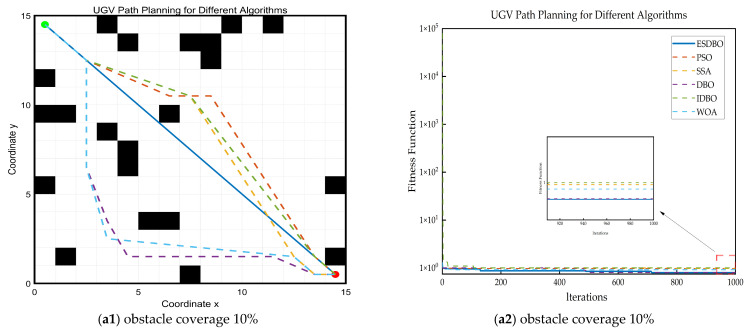

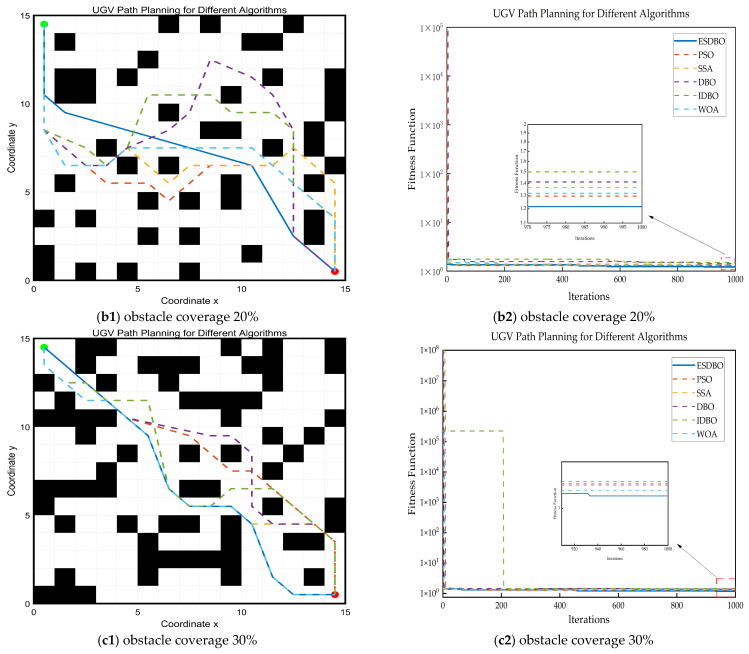
Path planning results and convergence curves on a 15 × 15 grid map.

**Figure 13:**
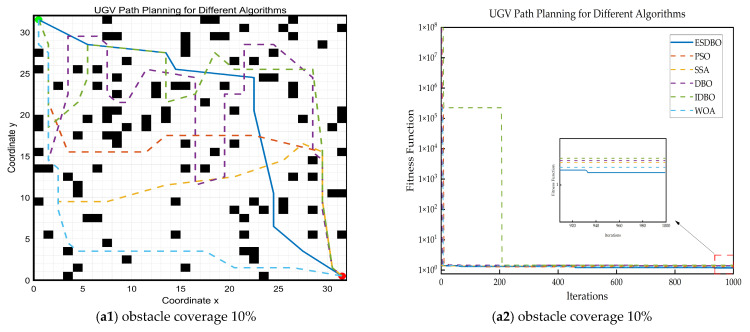

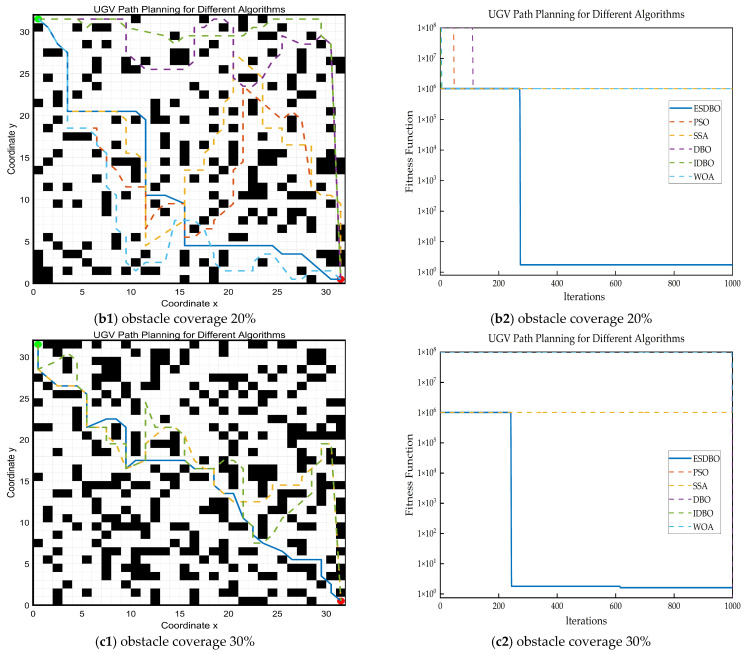
Path planning results and convergence curves on a 32 × 32 grid map.

The ESDBO algorithm was compared with five classical optimization algorithms, encompassing traditional meta-heuristic algorithms and an improved dung beetle optimization algorithm. The comparison set included the following: Particle Swarm Optimization (PSO), Whale Optimization Algorithm (WOA), the original Dung Beetle Optimization (DBO), Sparrow Search Algorithm (SSA), and an improved DBO algorithm (IDBO). Figure 11, Figure 12 and Figure 13 show the path trajectory.

Figure 11, Figure 12 and Figure 13 and Table 5 show that ESDBO can stably generate coherent and executable collision-free paths under different combinations of map scale and obstacle density, and its comprehensive performance is more robust and consistent than PSO, SSA, WOA, DBO, and IDBO. With the increase in map scale or obstacle density, this advantage is further highlighted.

In the 10 × 10 grid map, each algorithm can obtain a feasible solution, and the difference is mainly reflected in the geometric quality of the path. Figure 11 shows that some methods are prone to redundant turns and unnecessary detours, increasing the number of turns and a decrease in smoothness. In contrast, ESDBO maintains a shorter path and less steering while satisfying the feasibility, reflecting more effective local search and stronger convergence accuracy in the low-dimensional feasible region.

In the 15 × 15 grid map, as the obstacle density increases, the feasible space is significantly compressed, as shown in Figure 12. The comparison algorithm is more prone to trajectory fluctuations under high-density conditions, and the safety margin is reduced. ESDBO can maintain a more stable trajectory shape and generate a path closer to the direct path under low-density conditions, indicating that it has more reliable identification and utilization capabilities for key feasible channels in medium-sized scenarios.

In the 32 × 32 grid map, the robustness advantage of ESDBO is the most prominent. As shown in Figure 13, it is difficult for multiple comparison algorithms to obtain effective paths under narrow channels and strongly constrained feasible regions. ESDBO can still output collision-free solutions and take into account the dual goals of “feasibility and path quality” when the feasible region is highly fragmented.

To evaluate the actual advantages and limitations of the proposed algorithm more fairly, the traditional A* algorithm is widely considered the standard baseline for path planning based on raster maps. Therefore, we supplement the traditional 8-neighborhood A* algorithm and compare it with the proposed ESDBO under the same experimental settings. The comparison results are shown in the following Table 6.

As shown in Table 6, A* consistently achieves the lowest computational time in all feasible scenarios, which is expected due to its deterministic graph search property. In terms of path length, A * shows advantages in some medium–low density environments (e.g., 15 × 15 grids with 20% obstacle density), reflecting its theoretical optimality in searching the shortest path in static and fully known maps.

In contrast, in the grid map instance with high obstacle density, A* cannot find a feasible path. This result shows that A* is constrained by discrete space and fails to identify the feasible route, and the relevant index is recorded as Inf. In most test cases, the proposed ESDBO achieves a path length of the same order of magnitude as A*, and the generated path has significantly fewer turning points and better smoothness, indicating that it has stronger robustness in complex environments.

In summary, ESDBO achieves shorter paths and fewer turns in most scenarios and still maintains stable convergence and feasible solution output under high constraints, reflecting stronger global exploration ability and inhibition of premature convergence.

## 6. Conclusions

Aiming at the problems of low search efficiency, insufficient convergence accuracy, and ease of falling into local optimum in the path planning of UGV by DBO algorithm, an improved algorithm based on sine mapping, adaptive information volatilization mutation strategy and multi-mechanism co-evolution strategy ESDBO is proposed. Based on maintaining the computational complexity of the original DBO, the algorithm effectively enhances the global exploration ability and local convergence accuracy and significantly improves the stability of the solution in complex environments.

Through experimental verification on the CEC2017 benchmark function and 2D grid maps of different scales and different obstacle densities, the results show that ESDBO has faster convergence speed, higher solution accuracy, and stronger stability in solving high-dimensional optimization problems. Compared with the typical meta-heuristic algorithm and the improved DBO variant algorithm, the path length, safety margin and convergence performance are significantly improved. The algorithm has good scalability and real-time performance and can be extended to three-dimensional UGV trajectory planning, manipulator motion control, multi-agent collaborative optimization and other scenarios.

Future research will focus on two main directions. Firstly, the proposed framework is extended to a dynamic and uncertain 3D environment, incorporating environmental awareness and online re-planning mechanisms to deal with time-varying obstacles and constraints. Secondly, in addition to UGV path planning, the algorithm will be further adapted to other robot fields with similar optimization characteristics, such as UAV source seeking and target positioning, where implicit, noisy, and non-convex objective functions are usually encountered. Through these extensions, ESDBO is expected to evolve into a more general and versatile bio-inspired optimization framework for heterogeneous robot platforms and complex real-world scenarios.

## Figures and Tables

**Table 1:** Parameter variable setting.

Algorithms	Parameter Variable Value
PSO	Learning factor $C_{1}$ = $C_{2}$ = 2, initial inertia weight $W_{\max}$ = 0.9, inertia weight in maximum evolutionary selection $W_{\min}$ = 0.6
WOA	Random number a = [−1, 1]
GWO	The convergence factor a decreases linearly from 2 to 0 during substitution selection
WHO	Proportion of leading horses $P_{s}$ = 0.2, cross operation ratio $P_{c}$ = 0.13
DBO	Deviation factor *K* = 0.1, random number *b* = 0.3, *c* = 0.5
IDBO	Chaotic map parameter λ = 4, perturbation coefficient η = 0.5, adaptive parameter α = 0.9, local search probability P = 0.2
TDBO	Step length coefficient δ = 1.5, trajectory adjustment factor μ = 0.8, information sharing probability Ps = 0.1, random control factor r = [0, 1]
ESDBO	Minimum volatile factor $\rho_{\min}$ = 0, maximum volatile factor $\rho_{\max}$ = 1, chemotaxis step length *C*_*c*_ = 0.05

**Table 2:** Experimental results (dim = 30).

Function	Measure	PSO	WOA	GWO	WHO	DBO	IDBO	TDBO	ESDBO
F1	Average	2.46 × 10^0^	1.39 × 10^−73^	1.16 × 10^−27^	3.81 × 10^−43^	1.57 × 10^−107^	**0.00 × 10^0^**	**0.00 × 10^0^**	**0.0 × 10^0^**
	Std	1.1 × 10^0^	9.53 × 10^−73^	1.78 × 10^−27^	4.1 × 10^−42^	2.20 × 10^−106^	**0.00 × 10^0^**	**0.00 × 10^0^**	**0.0 × 10^0^**
F2	Average	1.10 × 10^1^	1.70 × 10^−49^	1.04 × 10^−16^	3.93 × 10^−25^	1.79 × 10^−47^	**0.00 × 10^0^**	**0.00 × 10^0^**	**0.0 × 10^0^**
	Std	7.61 × 10^0^	2.16 × 10^−48^	7.58 × 10^−17^	1.43 × 10^−24^	2.53 × 10^−46^	**6.28 × 10^−125^**	**0.00 × 10^0^**	**0.0 × 10^0^**
F3	Average	1.89 × 10^2^	4.58 × 10^4^	2.58 × 10^−5^	2.85 × 10^−24^	5.62 × 10^−17^	**0.00 × 10^0^**	1.59 × 10^−153^	3.03 × 10^−132^
	Std	5.56 × 10^1^	1.39 × 10^4^	1.07 × 10^−4^	3.23 × 10^−23^	7.95 × 10^−16^	**0.00 × 10^0^**	2.24 × 10^−152^	4.29 × 10^−131^
F4	Average	1.97 × 10^0^	4.89 × 10^1^	7.26 × 10^−7^	2.65 × 10^−17^	4.66 × 10^−46^	**1.82 × 10^−110^**	1.35 × 10^−94^	2.57 × 10^−69^
	Std	2.67 × 10^−1^	2.66 × 10^1^	6.91 × 10^−07^	1.14 × 10^−16^	4.91 × 10^−45^	**2.35 × 10^−109^**	1.90 × 10^−93^	3.624 × 10^−68^
F5	Average	9.36 × 10^2^	2.80 × 10^1^	2.71 × 10^1^	3.51 × 10^1^	**2.58 × 10^1^**	2.66 × 10^1^	2.86 × 10^1^	2.62 × 10^1^
	Std	5.15 × 10^2^	4.50 × 10^−1^	7.92 × 10^−1^	2.65 × 10^0^	**2.37 × 10^−1^**	5.19 × 10^0^	2.79 × 10^−1^	3.65 × 10^−1^
F6	Average	2.44 × 10^0^	3.58 × 10^−1^	7.64 × 10^−1^	**2.42 × 10^−1^**	4.44 × 10^−1^	2.87 × 10^−1^	4.13 × 10^0^	3.10 × 10^−1^
	Std	1.01 × 10^0^	2.11 × 10^−1^	4.15 × 10^−1^	5.78 × 10^−1^	9.12 × 10^−1^	3.09 × 10^−1^	3.63 × 10^−1^	**4.29 × 10^−2^**
F7	Average	7.46 × 10^0^	3.90 × 10^−3^	1.93 × 10^−3^	1.10 × 10^−3^	1.20 × 10^−4^	8.43 × 10^−4^	9.020 × 10^−4^	**3.68 × 10^−4^**
	Std	7.12 × 10^0^	4.47 × 10^−3^	9.81 × 10^−4^	9.04 × 10^−4^	1.14 × 10^−3^	8.31 × 10^−4^	7.77 × 10^−4^	**3.31 × 10^−4^**
F8	Average	−6.13 × 10^3^	**−1.03 × 10^4^**	−5.97 × 10^3^	−8.85 × 10^3^	−8.83 × 10^3^	−9.20 × 10^3^	−9.25 × 10^3^	−1.01 × 10^4^
	Std	1.31 × 10^3^	1.73 × 10^3^	8.81 × 10^2^	5.51 × 10^2^	1.86 × 10^3^	2.74 × 10^1^	**5.83 × 10^−1^**	1.90 × 10^3^
F9	Average	2.10 × 10^2^	5.44 × 10^−3^	3.05 × 10^0^	1.22 × 10^−9^	2.20 × 10^0^	**0.00 × 10^0^**	**0.00 × 10^0^**	**0.0 × 10^0^**
	Std	3.59 × 10^1^	7.70 × 10^−2^	3.90 × 10^0^	1.52 × 10^−8^	1.38 × 10^1^	**0.00 × 10^0^**	**0.00 × 10^0^**	**0.0 × 10^0^**
F10	Average	2.59 × 10^0^	3.62 × 10^−15^	1.02 × 10^−13^	1.26 × 10^−15^	**4.44 × 10^−16^**	**4.44 × 10^−16^**	4.62 × 10^−16^	**4.44 × 10^−16^**
	Std	4.37 × 10^−1^	2.60 × 10^−15^	1.89 × 10^−14^	1.48 × 10^−15^	2.51 × 10^−16^	**0.00 × 10^0^**	2.51 × 10^−16^	**0.00 × 10^0^**
F11	Average	1.23 × 10^−1^	4.20 × 10^−3^	4.67 × 10^−3^	**0.0 × 10^0^**	7.78 × 10^−4^	**0.00 × 10^0^**	**0.00 × 10^0^**	**0.0 × 10^0^**
	Std	4.79 × 10^−2^	2.35 × 10^−2^	9.82 × 10^−3^	**0.0 × 10^0^**	8.53 × 10^−3^	**0.00 × 10^0^**	**0.00 × 10^0^**	**0.0 × 10^0^**
F12	Average	4.56 × 10^−2^	2.91 × 10^−2^	4.28 × 10^−2^	2.62 × 10^−2^	**1.50 × 10^−3^**	8.44 × 10^−3^	4.39 × 10^−1^	7.82 × 10^−3^
	Std	4.65 × 10^−2^	3.61 × 10^−2^	2.08 × 10^−2^	3.6 × 10^−1^	**2.52 × 10^−3^**	1.19 × 10^−2^	9.68 × 10^−2^	2.67 × 10^−3^
F13	Average	5.87 × 10^−1^	4.84 × 10^−1^	6.57 × 10^−1^	6.20 × 10^−2^	6.50 × 10^−1^	8.54 × 10^−2^	2.69 × 10^0^	**5.07 × 10^−2^**
	Std	2.58 × 10^−1^	2.42 × 10^−1^	2.50 × 10^−1^	4.49 × 10^−2^	8.54 × 10^−1^	1.12 × 10^−1^	1.03 × 10^−1^	**2.50 × 10^−2^**
F14	Average	3.13 × 10^0^	2.70 × 10^0^	4.27 × 10^0^	**1.36 × 10^0^**	1.47 × 10^0^	2.83 × 10^0^	2.77 × 10^0^	1.90 × 10^0^
	Std	2.49 × 10^0^	2.96 × 10^0^	4.06 × 10^0^	**1.22 × 10^0^**	1.65 × 10^0^	3.11 × 10^0^	1.99 × 10^0^	1.27 × 10^0^
F15	Average	5.50 × 10^−3^	7.49 × 10^−4^	4.49 × 10^−3^	2.86 × 10^−3^	7.93 × 10^−4^	**3.50 × 10^−4^**	6.11 × 10^−4^	5.88 × 10^−4^
	Std	7.94 × 10^−3^	5.72 × 10^−4^	8.65 × 10^−3^	6.19 × 10^−3^	3.77 × 10^−4^	**6.00 × 10^−5^**	3.98 × 10^−4^	2.30 × 10^−4^
F16	Average	−1.0 × 10^0^	−1.0 × 10^0^	−1.0 × 10^0^	−1.0 × 10^0^	−1.0 × 10^0^	**−1.03 × 10^0^**	**−1.03 × 10^0^**	**−1.03 × 10^0^**
	Std	4.24 × 10^−16^	9.85 × 10^−10^	8.81 × 10^−6^	9.69 × 10^−6^	**3.30 × 10^−16^**	2.71 × 10^−5^	2.82 × 10^−5^	4.13 × 10^−16^
F17	Average	**4.0 × 10^−1^**	**4.0 × 10^−1^**	**4.0 × 10^−1^**	**4.0 × 10^−1^**	**4.0 × 10^−1^**	3.98 × 10^−1^	3.98 × 10^−1^	**4.0 × 10^−1^**
	Std	**3.34 × 10^−16^**	2.19 × 10^−5^	1.36 × 10^−4^	4.45 × 10^−5^	1.40 × 10^−6^	2.85 × 10^−5^	2.85 × 10^−4^	**3.34 × 10^−16^**
F18	Average	**3.0 × 10^0^**	**3.0 × 10^0^**	4.62 × 10^0^	3.14 × 10^0^	3.13 × 10^0^	3.15 × 10^0^	3.07 × 10^0^	**3.0 × 10^0^**
	Std	**4.91 × 10^−15^**	6.34 × 10^−4^	1.14 × 10^0^	5.74 × 10^−3^	1.91 × 10^0^	1.91 × 10^0^	8.69 × 10^−2^	5.83 × 10^−15^
F19	Average	−5.76 × 10^0^	−5.77 × 10^0^	**−5.79 × 10^0^**	**−5.79 × 10^0^**	−5.76 × 10^0^	−5.58 × 10^0^	−5.76 × 10^0^	**−5.79 × 10^0^**
	Std	5.23 × 10^−2^	9.65 × 10^−2^	1.89 × 10^−02^	2.06 × 10^−6^	4.89 × 10^−2^	3.39 × 10^−1^	5.19 × 10^−2^	**8.90 × 10^−16^**
F20	Average	−3.34 × 10^0^	−3.30 × 10^0^	−3.46 × 10^0^	−3.49 × 10^0^	−3.43 × 10^0^	−3.02 × 10^0^	−3.33 × 10^0^	**−3.50 × 10^0^**
	Std	2.32 × 10^−1^	2.07 × 10^−1^	1.23 × 10^−1^	1.76 × 10^−1^	2.10 × 10^−1^	6.80 × 10^−1^	1.83 × 10^−1^	**1.67 × 10^−2^**
F21	Average	−9.54 × 10^0^	−6.99 × 10^0^	−1.04 × 10^1^	−8.25 × 10^0^	−8.72 × 10^0^	−5.13 × 10^0^	−7.96 × 10^0^	**−1.05 × 10^1^**
	Std	2.36 × 10^0^	3.32 × 10^0^	1.06 × 10^0^	3.35 × 10^0^	2.78 × 10^0^	2.50 × 10^0^	2.27 × 10^0^	**5.39 × 10^−1^**
F22	Average	−9.38 × 10^0^	−6.76 × 10^0^	−1.02 × 10^1^	−8.24 × 10^0^	−8.66 × 10^0^	−5.39 × 10^0^	−7.93 × 10^0^	**−1.05 × 10^1^**
	Std	2.59 × 10^0^	3.32 × 10^0^	1.43 × 10^0^	3.46 × 10^0^	2.76 × 10^0^	2.70 × 10^0^	2.26 × 10^0^	**5.43 × 10^−2^**
F23	Average	−9.83 × 10^0^	−6.99 × 10^0^	−1.02 × 10^1^	−7.78 × 10^0^	−8.59 × 10^0^	−5.14 × 10^0^	−8.03 × 10^0^	**−1.04 × 10^1^**
	Std	2.08 × 10^0^	3.26 × 10^0^	**1.48 × 10**	3.51 × 10^0^	2.79 × 10^0^	2.44 × 10^0^	2.22 × 10^0^	6.93 × 10^−1^
Avg MeanRank		5.71	5.26	5.22	4.13	3.37	4.31	5.00	2.98
Holm post−hoc (+/=/−)		17/2/4	17/5/1	17/1/5	12/3/8	10/5/8	11/6/6	15/6/2	−

**Table 3:** Experimental results (dim = 50).

Function	Measure	PSO	WOA	GWO	WHO	DBO	IDBO	TDBO	ESDBO
F1	Average	1.76 × 10^0^	3.56 × 10^−82^	2.44 × 10^−33^	1.04 × 10^−49^	1.02 × 10^−113^	**0.00 × 10^0^**	**0.00 × 10^0^**	**0.00 × 10^0^**
	Std	8.37 × 10^−1^	3.75 × 10^−81^	5.93 × 10^−33^	5.68 × 10^−49^	1.28 × 10^−112^	**0.00 × 10^0^**	**0.00 × 10^0^**	**0.00 × 10^0^**
F2	Average	8.83 × 10^0^	2.86 × 10^−51^	6.81 × 10^−20^	2.07 × 10^−28^	5.07 × 10^−51^	**0.00 × 10^0^**	**0.00 × 10^0^**	**0.00 × 10^0^**
	Std	7.67 × 10^0^	4.03 × 10^−50^	5.50 × 10^−20^	1.04 × 10^−27^	5.11 × 10^−50^	**0.00 × 10^0^**	**0.00 × 10^0^**	**0.00 × 10^0^**
F3	Average	1.23 × 10^2^	2.87 × 10^4^	6.91 × 10^−8^	1.56 × 10^−27^	9.53 × 10^−28^	**0.00 × 10^0^**	2.21 × 10^−164^	2.31 × 10^−140^
	Std	3.60 × 10^1^	1.07 × 10^4^	3.90 × 10^−7^	1.52 × 10^−26^	1.03 × 10^−26^	**0.00 × 10^0^**	**0.00 × 10^0^**	3.21 × 10^−139^
F4	Average	1.80 × 10^0^	3.72 × 10^1^	2.27 × 10^−8^	1.18 × 10^−19^	5.91 × 10^−51^	**8.14 × 10^−117^**	2.81 × 10^−104^	9.98 × 10^−71^
	Std	2.29 × 10^−1^	2.88 × 10^1^	3.47 × 10^−8^	3.15 × 10^−19^	7.20 × 10^−53^	**8.24 × 10^−116^**	3.98 × 10^−103^	1.36 × 10^−69^
F5	Average	7.56 × 10^2^	2.75 × 10^1^	2.67 × 10^1^	2.68 × 10^1^	4.92 × 10^1^	**2.64 × 10^1^**	2.83 × 10^1^	2.83 × 10^1^
	Std	4.81 × 10^2^	4.50 × 10^−1^	7.18 × 10^−1^	8.28 × 10^0^	1.52 × 10^1^	5.86 × 10^0^	**2.76 × 10^−1^**	3.32 × 10^−1^
F6	Average	1.72 × 10^0^	1.20 × 10^0^	2.70 × 10^0^	4.60 × 10^−1^	**2.73 × 10^−1^**	9.20 × 10^−1^	3.65 × 10^0^	5.07 × 10^−1^
	Std	8.06 × 10^−1^	1.22 × 10^0^	1.93 × 10^0^	1.85 × 10^0^	6.09 × 10^−1^	1.14 × 10^0^	4.08 × 10^−1^	**6.84 × 10^−2^**
F7	Average	5.04 × 10^0^	2.13 × 10^−3^	1.27 × 10^−3^	8.32 × 10^−4^	1.94 × 10^−3^	6.54 × 10^−4^	9.91 × 10^−3^	**2.27 × 10^−4^**
	Std	4.50 × 10^0^	2.40 × 10^−3^	7.03 × 10^−4^	5.96 × 10^−4^	1.71 × 10^−3^	4.97 × 10^−5^	7.01 × 10^−5^	**1.84 × 10^−5^**
F8	Average	−6.69 × 10^3^	−1.10 × 10^4^	−6.03 × 10^3^	−9.20 × 10^3^	−9.09 × 10^3^	−1.26 × 10^4^	−1.26 × 10^4^	**−1.53 × 10^4^**
	Std	8.53 × 10^2^	1.60 × 10^3^	7.74 × 10^2^	4.78 × 10^2^	1.31 × 10^3^	1.84 × 10^1^	**3.67 × 10^−1^**	2.31 × 10^3^
F9	Average	1.95 × 10^2^	3.13 × 10^−15^	1.81 × 10^0^	8.61 × 10^−14^	9.77 × 10^0^	**0.00 × 10^0^**	**0.00 × 10^0^**	**0.0 × 10^0^**
	Std	3.42 × 10^1^	1.53 × 10^−14^	3.20 × 10^0^	9.43 × 10^−13^	3.24 × 10^10^	**0.00 × 10^0^**	**0.00 × 10^0^**	**0.0 × 10^0^**
F10	Average	2.25 × 10^0^	3.83 × 10^−15^	4.27 × 10^−14^	7.81 × 10^−16^	4.62 × 10^−16^	**4.44 × 10^−16^**	**4.44 × 10^−16^**	**4.44 × 10^−16^**
	Std	4.75 × 10^−1^	2.23 × 10^−15^	5.03 × 10^−15^	1.04 × 10^−15^	2.51 × 10^−16^	**0.00 × 10^0^**	2.51 × 10^−16^	**0.00 × 10^0^**
F11	Average	9.44 × 10^−2^	7.90 × 10^−3^	4.19 × 10^−3^	**0.0 × 10^0^**	6.97 × 10^−4^	**0.00 × 10^0^**	**0.00 × 10^0^**	**0.0 × 10^0^**
	Std	4.34 × 10^−2^	3.16 × 10^−2^	8.67 × 10^−3^	**0.0 × 10^0^**	6.32 × 10^−3^	**0.00 × 10^0^**	**0.00 × 10^0^**	**0.0 × 10^0^**
F12	Average	3.11 × 10^−2^	2.03 × 10^−2^	2.89 × 10^−1^	8.81 × 10^−3^	**6.56 × 10^−4^**	3.87 × 10^−2^	3.57 × 10^−1^	7.60 × 10^−3^
	Std	4.18 × 10^−2^	1.61 × 10^−1^	1.58 × 10^−2^	3.57 × 10^−2^	7.39 × 10^−3^	**5.13 × 10^−3^**	6.97 × 10^−2^	2.81 × 10^−4^
F13	Average	4.04 × 10^−1^	1.89 × 10^−1^	3.61 × 10^−1^	1.88 × 10^−2^	1.06 × 10^−1^	7.09 × 10^−2^	2.56 × 10^0^	**4.29 × 10^−2^**
	Std	1.74 × 10^−1^	1.31 × 10^−1^	1.80 × 10^−1^	5.46 × 10^−2^	1.34 × 10^−1^	8.13 × 10^−2^	1.60 × 10^−1^	**1.91 × 10^−2^**
Avg MeanRank		7.35	5.06	5.96	3.77	3.99	2.93	4.08	2.86
Holm post−hoc (+/=/−)		13/0/0	10/0/3	11/0/2	6/2/5	8/2/3	5/5/3	5/6/2	−

**Table 4:** Experimental results (dim = 100).

Function	Measure	PSO	WOA	GWO	WHO	DBO	IDBO	TDBO	ESDBO
F1	Average	1.01 × 10^0^	3.42 × 10^−95^	6.60 × 10^−41^	7.79 × 10^−59^	6.45 × 10^−105^	**0.00 × 10^0^**	3.70 × 10^−205^	**0.00 × 10^0^**
	Std	4.80 × 10^0^	3.93 × 10^−94^	1.37 × 10^−40^	7.40 × 10^−58^	9.12 × 10^−104^	**0.00 × 10^0^**	**0.00 × 10^0^**	**0.00 × 10^0^**
F2	Average	5.18 × 10^0^	1.74 × 10^−56^	5.46 × 10^−24^	2.69 × 10^−34^	1.60 × 10^−61^	**0.00 × 10^0^**	5.18 × 10^−105^	**0.00 × 10^0^**
	Std	4.65 × 10^0^	1,11 × 10^−55^	4.57 × 10^−24^	1.34 × 10^−33^	2.25 × 10^−60^	**0.00 × 10^0^**	7.91 × 10^0^	**0.00 × 10^0^**
F3	Average	7.79 × 10^1^	1.60 × 10^4^	1.67 × 10^−11^	2.70 × 10^−34^	1.10 × 10^−35^	**0.00 × 10^0^**	1.66 × 10^−182^	**0.00 × 10^0^**
	Std	1.86 × 10^1^	7.77 × 10^4^	5.77 × 10^−11^	1.52 × 10^−33^	1.56 × 10^−34^	**0.00 × 10^0^**	**0.00 × 10^0^**	**0.00 × 10^0^**
F4	Average	1.56 × 10^0^	2.57 × 10^1^	1.89 × 10^−10^	5.46 × 10^−23^	3.86 × 10^−52^	5.71 × 10^−98^	**1.17 × 10^−122^**	1.31 × 10^−75^
	Std	1.56 × 10^0^	2.57 × 10^1^	1.94 × 10^−10^	2.99 × 10^−22^	5.31 × 10^−51^	5.59 × 10^−97^	**1.65 × 10^−121^**	1.30 × 10^−74^
F5	Average	5.4 × 10^3^	9.7 × 10^1^	9.6 × 10^1^	1.2 × 10^2^	9.5 × 10^1^	**2.67 × 10^1^**	2.82 × 10^1^	2.87 × 10^1^
	Std	4.70 × 10^2^	6.15 × 10^−1^	9.77 × 10^0^	1.36 × 10^1^	1.17 × 10^1^	6.82 × 10^0^	**1.92 × 10^−1^**	3.27 × 10^−1^
F6	Average	1.97 × 10^0^	4.86 × 10^0^	9.81 × 10^0^	7.55 × 10^0^	4.63 × 10^0^	1.17 × 10^−1^	3.51 × 10^0^	**2.01 × 10^−1^**
	Std	4.83 × 10^−1^	2.14 × 10^0^	9.78 × 10^0^	6.05 × 10^−1^	8.99 × 10^0^	1.55 × 10^−1^	**4.41 × 10^−1^**	1.11 × 10^0^
F7	Average	3.25 × 10^0^	3.86 × 10^−3^	7.65 × 10^−3^	1.45 × 10^−3^	2.06 × 10^−3^	1.17 × 10^−3^	1.12 × 10^−3^	**3.88 × 10^−5^**
	Std	3.26 × 10^0^	4.25 × 10^−3^	4.23 × 10^−3^	8.27 × 10^−4^	1.72 × 10^−3^	9.85 × 10^−4^	7.47 × 10^−4^	**3.59 × 10^−5^**
F8	Average	−3.79 × 10^3^	−1.17 × 10^4^	−6.48 × 10^3^	−9.56 × 10^3^	−9.35 × 10^3^	−1.26 × 10^4^	−1.26 × 10^4^	**−1.44 × 10^4^**
	Std	7.72 × 10^2^	1.26 × 10^3^	7.50 × 10^2^	4.77 × 10^2^	8.75 × 10^2^	1.08 × 10^2^	**4.59 × 10^−1^**	2.39 × 10^3^
F9	Average	1.73 × 10^2^	**0.00 × 10^0^**	9.57 × 10^−1^	**0.00 × 10^0^**	2.17 × 10^1^	**0.00 × 10^0^**	**0.00 × 10^0^**	**0.00 × 10^0^**
	Std	3.35 × 10^1^	4.02 × 10^−15^	2.17 × 10^0^	**0.00 × 10^0^**	4.48 × 10^1^	**0.00 × 10^0^**	**0.00 × 10^0^**	**0.00 × 10^0^**
F10	Average	1.88 × 10^0^	4.05 × 10^−15^	2.70 × 10^−14^	4.62 × 10^−16^	**4.44 × 10^−16^**	**4.44 × 10^−16^**	**4.44 × 10^−16^**	**4.44 × 10^−16^**
	Std	4.88 × 10^−1^	2.35 × 10^−15^	3.61 × 10^−15^	2.51 × 10^−16^	**0.00 × 10^0^**	**0.00 × 10^0^**	**0.00 × 10^0^**	**0.00 × 10^0^**
F11	Average	6.00 × 10^−2^	3.28 × 10^−3^	2.63 × 10^−3^	**0.00 × 10^0^**	1.92 × 10^−3^	**0.00 × 10^0^**	**0.00 × 10^0^**	**0.00 × 10^0^**
	Std	3.02 × 10^−2^	1.31 × 10^−2^	6.06 × 10^−3^	**0.00 × 10^0^**	1.37 × 10^2^	**0.00 × 10^0^**	**0.00 × 10^0^**	**0.00 × 10^0^**
F12	Average	1.69 × 10^−2^	1.39 × 10^−2^	1.58 × 10^−2^	3.11 × 10^−3^	**5.57 × 10^−4^**	4.67 × 10^−3^	3.98 × 10^−1^	7.23 × 10^−3^
	Std	2.54 × 10^−2^	3.84 × 10^−3^	1.05 × 10^−2^	1.77 × 10^−2^	7.34 × 10^−3^	**7.45 × 10^−3^**	7.60 × 10^−2^	1.41 × 10^−3^
F13	Average	2.51 × 10^−1^	3.57 × 10^−2^	2.02 × 10^−1^	5.36 × 10^0^	3.35 × 10^−2^	7.29 × 10^−2^	2.34 × 10^0^	**2.65 × 10^−2^**
	Std	1.22 × 10^−1^	3.83 × 10^−2^	1.43 × 10^−1^	2.28 × 10^0^	5.75 × 10^−2^	1.01 × 10^−1^	3.32 × 10^−1^	**5.62 × 10^−3^**
Avg MeanRank		7.18	5.01	6.08	4.71	3.91	2.75	3.64	2.71
Holm post−hoc (+/=/−)		13/0/0	9/2/2	12/0/1	8/4/1	8/3/2	3/7/3	6/3/4	−

**Table 5:** Statistical summary of test results.

Map Size	Obstacle Density	Metric	PSO	SSA	WOA	DBO	IDBO	ESDBO
10 × 10	10%	Path Length	12.893	12.893	12.893	13.893	12.893	12.893
		Turns	2	2	2	3	2	2
		Smoothness (norm)	0.3936	0.3936	0.3936	0.7581	0.3936	0.3936
		Safety (norm)	1.0390	1.0390	1.0390	0.9091	1.0390	1.0390
		Execution time	10.7544	6.0053	9.5469	3.6691	4.8184	6.1826
	20%	Path Length	13.648	14.463	13.543	13.543	14.136	13.543
		Turns	3	4	2	2	3	2
		Smoothness (norm)	0.6057	0.8634	0.3916	0.3916	0.5737	0.3916
		Safety (norm)	1.3636	1.4141	1.3636	1.3636	1.4141	1.3636
		Execution time	5.878	5.5711	5.3130	3.812	5.5712	4.6446
	30%	Path Length	15.071	16.550	14.485	15.071	14.893	14.485
		Turns	5	5	4	5	7	4
		Smoothness (norm)	1.250	0.7302	0.8333	1.250	1.0531	0.8333
		Safety (norm)	1.8182	1.8182	1.8182	1.8182	1.8182	1.8182
		Execution time	3.8966	2.2847	3.7515	2.7515	3.6860	3.2000
15 × 15	10%	Path Length	21.010	24.641	20.563	19.899	24.641	19.799
		Turns	4	4	5	1	5	0
		Smoothness (norm)	1.1286	1.3333	0.799	0.2409	0.2409	0.0000
		Safety (norm)	1.2091	1.1364	1.0390	1.2091	1.0390	1.2121
		Execution time	9.4258	9.0330	15.4965	5.1271	9.50268	9.4691
	20%	Path Length	28.728	28.728	26.307	32.770	30.884	22.202
		Turns	6	7	6	11	11	4
		Smoothness (norm)	0.7692	1.0256	1.1111	1.2500	1.6667	0.7143
		Safety (norm)	1.5141	1.5909	1.5223	1.4876	1.6667	1.4121
		Execution time	5.3364	2.5559	3.7772	3.5370	2.2614	4.9971
	30%	Path Length	21.890	23.062	22.224	23.608	24.999	21.638
		Turns	8	10	10	11	11	7
		Smoothness (norm)	0.9206	1.2751	1.1784	1.3889	1.4740	0.8123
		Safety (norm)	1.6970	1.6883	1.5584	1.6883	1.6970	1.5385
		Execution time	4.9243	2.6887	4.9243	3.5006	3.7994	4.8526
32 × 32	10%	Path Length	59.324	67.150	57.070	122.779	83.532	51.632
		Turns	9	12	12	22	10	7
		Smoothness (norm)	1.5000	1.4690	1.2914	2.2097	2.0490	1.3661
		Safety (norm)	1.6189	1.7576	1.6818	1.8556	1.8556	1.3636
		Execution time	21.6357	14.0402	13.1589	9.3737	18.2879	17.0407
	20%	Path Length	Inf	Inf	Inf	Inf	Inf	67.507
		Turns	25	31	28	20	35	25
		Smoothness (norm)	Inf	Inf	Inf	Inf	Inf	1.7241
		Safety (norm)	Inf	Inf	Inf	Inf	Inf	1.7009
		Execution time	2.3780	3.17030	28.4463	0.7765	18.0420	17.6111
	30%	Path Length	Inf	Inf	Inf	Inf	Inf	56.605
		Turns	Inf	Inf	Inf	Inf	26	26
		Smoothness (norm)	Inf	Inf	Inf	Inf	Inf	1.8026
		Safety (norm)	Inf	Inf	Inf	Inf	Inf	1.7532
		Execution time	Inf	Inf	Inf	Inf	Inf	17.7813

**Table 6:** Comparison results with the A* algorithm.

Map Size	Obstacle Density	Algorithm	Path Length	Turn	Smoothness (Norm)	Safety (Norm)	Time
10 × 10	10%	ESDBO	12.893	2	0.3936	1.0390	6.1826
		A*	13.071	1	0.4538	1.0390	0.3619
	20%	ESDBO	13.543	2	0.3916	1.3636	4.6446
		A*	13.877	4	0.8634	1.2987	0.4090
	30%	ESDBO	14.485	4	0.8333	1.8182	3.2000
		A*	Inf	Inf	Inf	Inf	0.0007
15 × 15	10%	ESDBO	19.799	0	0	1.2121	9.4691
		A*	20.385	3	5	1.0390	0.2121
	20%	ESDBO	22.202	4	0.7143	1.4121	4.9971
		A*	20.385	7	1.3730	1.6364	0.3718
	30%	ESDBO	21.638	7	0.8123	1.5385	4.8526
		A*	23.595	10	1.9194	1.6883	0.4142
32 × 32	10%	ESDBO	51.632	7	1.3661	1.3636	17.0407
		A*	50.188	25	1.7031	1.7555	0.7282
	20%	ESDBO	54.307	6	1.2124	0.7009	14.6111
		A*	52.643	10	1.2357	1.3333	0.6024
	30%	ESDBO	56.605	26	1.8630	1.8182	17.7813
		A*	Inf	Inf	Inf	Inf	0.0025

## Data Availability

The data presented in this study are available on request from the corresponding author.
